# Perhexiline maleate enhances antitumor efficacy of cisplatin in neuroblastoma by inducing over-expression of NDM29 ncRNA

**DOI:** 10.1038/srep18144

**Published:** 2015-12-17

**Authors:** Serena Vella, Ilaria Penna, Luca Longo, Giulia Pioggia, Patrizia Garbati, Tullio Florio, Fabio Rossi, Aldo Pagano

**Affiliations:** 1 Dept. of Experimental Medicine (DIMES), University of Genova, Genova, Italy; 2 IRCCS-AOU San Martino-IST, Genova, Italy; 3 Sect. of Pharmacology, Dept. of Internal Medicine (DiMI) and Center of Excellence for Biomedical Research (CEBR), University of Genova, Genova, Italy; 4 The Biomedical Research Centre, University of British Columbia, Vancouver, British Columbia V6T 1Z3, Canada; 5 Department of Medical Genetics, University of British Columbia, Vancouver, British Columbia V6T 1Z3, Canada

## Abstract

High Risk Neuroblastoma (HR-NB) is a pediatric cancer characterized by high malignancy and remarkable cell heterogeneity within the tumour nodules. In a recent study, we demonstrated that *in vitro* and *in vivo* over-expression of the non-coding RNA NDM29 (neuroblastoma differentiation marker 29) induces NB cell differentiation, dramatically reducing their malignancy. Among gene expression changes, differentiated phenotype induced by NDM29 is characterized by decrease of the expression of ABC transporters responsible for anticancer drug resistance. Thus, the pharmacological induction of NDM29, in principle, might represent a possible novel strategy to increase cytotoxic drug responses. In this work, we identify a small molecule able to induce the expression of NDM29 in NB cells, conferring to malignant cells increased susceptibility to cisplatin cytotoxic effects. We demonstrate that the pharmacological induction of NDM29 expression *in vivo* enhances the antitumoral effects of chemotherapy specifically on tumour initiating/cancer stem cells sub-population, usually refractory to therapies and responsible for tumour relapse. In summary, we suggest a novel therapeutical approach possibly useful to treat very aggressive NB cases with poor prognosis. This novel pharmacological strategy aims to promote differentiation of “stem-like” cells to render them more susceptible to the killing action of cytotoxic anticancer drugs.

Neuroblastoma (NB) is a childhood tumour of the sympathetic nervous system characterized by a broad spectrum of clinical outcomes, ranging from spontaneous regression to fatal outcome despite aggressive therapies^1^.

NB is a disease of the adrenal lineage of the neural crest, derived from progenitor cells of the sympathetic nervous system^1^. Cellular heterogeneity is a hallmark of human NB, and cell lines established from several human NBs retain similar heterogeneity. Despite recent advancement in the understanding of its biological and molecular genetics features, NB still accounts for about 15% of all pediatric cancer deaths^2^,^3^. Innovative treatment approaches are therefore needed for this disease.

Based on consensus sequences for regulatory elements, we identified several putative Pol III promoters driving the expression of non coding RNAs (ncRNAs)^4^. One of these ncRNAs, named neuroblastoma differentiation marker 29 (NDM29)^5^, maps in intron 1 of the Achaete Scute-Like homologue 3 gene (ASCL3; RefSeq: NM_020646.1), in a 11p15.3 chromosomal region associated with oncosuppressive activity and whose genetic alterations are involved in NB development^6^,^7^.

Stable over-expression of NDM29 ncRNA is sufficient to induce NB cell differentiation, dramatically restricting their tumorigenic potential^5^.

Interestingly, we observed that the absolute level of expression of NDM29 RNA can be considered low and tightly regulated as its upregulation of 2.4-fold has such remarkable effects on cell phenotype^5^ and that the 350-fold increase (based on a relative and not absolute quantitation of this transcript by Real Time RT-PCR) observed in NB cells when compared to HeLa cells has to be ascribed to a very very low amount of this RNA in HeLa cells^5^.

Indeed, we have recently demonstrated that NDM29 over-expression drives human SKNBE2 NB cells to differentiate acquiring neuron-like traits, including (a) neuronal morphology endowed with well-distinguishable network of neuritic processes, (b) low proliferation rate, (c) excitatory properties associated to functional synapses, (d) reduction of stemness marker expression, (e) acquisition of anchorage-dependent growth, and (f) expression of specific neuronal markers^5^,^8^,^9^,^10^,^11^.

Moreover, we demonstrated that NDM29 over-expression actively prevents NB cell tumorigenicity *in vivo:* the number of tumour nodules developed in mice injected with the human NB cells SKNBE2 is inversely correlated with NDM29 expression, which leads to a significant decrease in tumour-initiating/stem-like cells^5^.

Interestingly, in these experiments we found that NB cells over-expressing NDM29 ncRNA show increased susceptibility to the effects of anticancer drugs commonly used in NB therapy to induce cell differentiation (i.e. cisplatin and doxorubicin), a process associated with inhibition of MDR1 expression (one of the key actors of chemoresistance)^5^.

In addition, we showed that the effects of NDM29 expression were at least in part due to a reduction in tumour initiating cell (TIC) content, suggesting that the pharmacological induction of NDM29 expression could represent a novel strategy to potentiate cisplatin cytotoxicity, extending the effects of this drug to normally refractory cancer cell subpopulations.

In the present study we used the “SOSA Approach” (Selective Optimization of Side Activities^12^) to identify the small molecule perhexiline as a drug able to promote NDM29 ncRNA expression and thus leading to *in vitro* and *in vivo* enhancement of the antitumoral effects of cisplatin. We further show that, in a mouse model of NB, the combined therapy with perhexiline/cisplatin reduces the growth rate of NB nodules and increases overall survival, suggesting that the pharmacological modulation of NDM29 expression represents a new tool to potentiate the effects of chemotherapeutics.

## Results

### NDM29-dependent down-regulation of ABC transporters in NB cells

We previously demonstrated that over-expression of NDM29 leads to the down-regulation of MDR1 (multidrug resistance protein 1; RefSeq NM_000927) expression, conferring to NB cells an increased susceptibility to the effects of cisplatin and doxorubicin^5^. In this study, we investigated whether the increased susceptibility was possibly ascribed to the down-regulation of other ABC transporters, so that over-expressing NDM29 ncRNA, multiple drugs might increase potency. We used the commercially available “Human drug transporter RT^2^ profiler PCR array” (SABiosciences) to profile the expression of 84 human transporter genes thought to play key roles in the resistance of cancer cells to chemotherapeutics (http://saweb2.sabiosciences.com/rt_pcr_product/HTML/PAHS-070A.html). We generated drug transporter expression profiles from, two SKNBE2 lines, mock (M) cells (transfected with empty vector) natively expressing the NDM29 ncRNA at basal level, and Stable 1 (S1) cells (transfected with NDM29 carrying vector) expressing 5.4 fold more NDM29 ncRNA.

**Figure 1:**
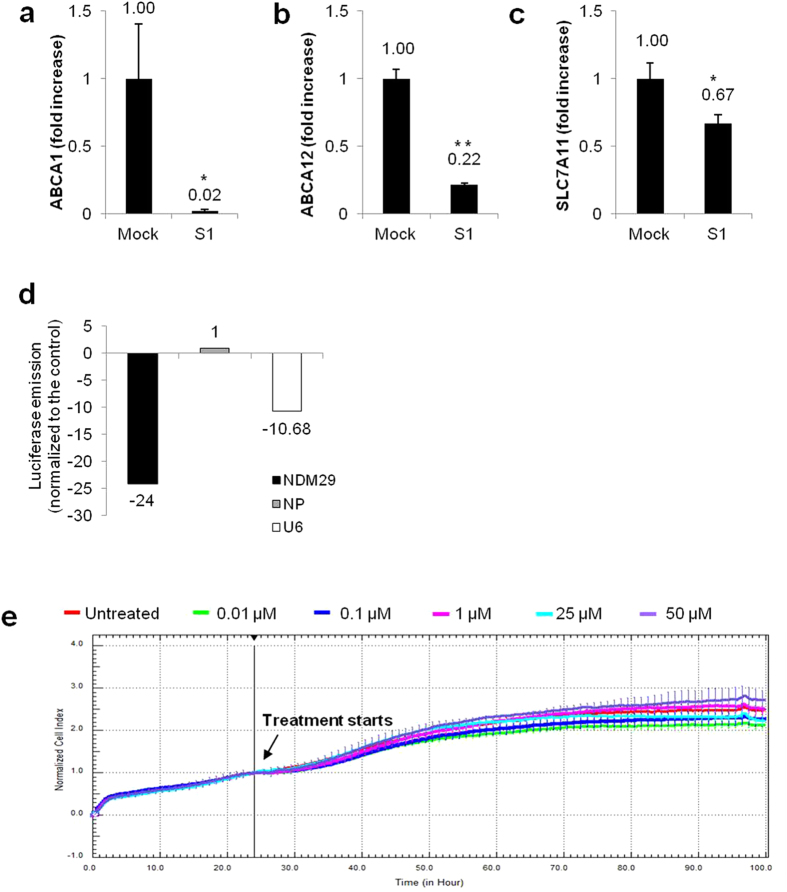
NDM29 induces the down-regulation of ABC transporters in NB cells and its expression is pharmacologically induced by perhexiline maleate. Expression levels of ABCA1 ((**a**) p < 0.0001), ABCA12 ((**b**) p < 0.0001) and SLC7A11 ((**c**) p = 0.0113) in Mock and S1 cells, measured by Real-time RT-PCR. One star (*) indicates p < 0.05, while two stars (**) indicate p < 0.001, two-tailed Student’s test. Results of the primary screening for perhexiline maleate (**d**). Results are reported as luciferase emission of treated *vs.* untreated cells. Luminescent signal is normalized to amounts of GPF of cells, for normalization of transfection efficiency. Effect of perhexiline (0.01-0.1-1-25-50 μM) (**e**) on the viability of SH-SY5Y wt was measured based on the dose–response curves of the cell index by the xCELLigence system. Cell Index (CI) was recorded every 30 minutes. Each trace at each concentration was an average of three replicates. Vehicle (DMSO): red curve; Perhexiline 0.01 μM: green curve; Perhexiline 0.1 μM: blue curve; Perhexiline 1 μM: pink curve; Perhexiline 25 μM: cyan curve; Perhexiline 50 μM: violet curve.

We found that NDM29 over-expression induced a generalized decrease in ABC transporter\expression, with 82 mRNAs down-regulated and only 2 up-regulated (see Supplementary Fig. S1). By arbitrarily imposing a cut-off value (with fold changes down to −50), we selected 3 genes whose expression was significantly modulated by NDM29 over-expression: ABCA1, ABCA12 and SLC7A11^13^,^14^,^15^,^16^,^17^. For these genes, expression changes observed using the array were individually validated by Real-Time RT-PCR (Fig. 1a–c, p < 0.0001, p < 0.0001 and p = 0.0113, respectively, compared to M cells).

Altogether these results suggest that NDM29 over-expression may lead to increased susceptibility to anticancer therapeutics by reducing the expression of multiple MDR transporters.

### Pharmacological induction of NDM29 ncRNA expression by perhexiline maleate

These data and results from our previous work^5^ strongly suggest that the up-regulation of endogenous NDM29 may have therapeutic value, prompting us to identify small molecule inducers of NDM29 ncRNA using the “SOSA Approach“^12^. Because SKNBE cells are resistant to cisplatin treatment^18^,^19^, we decided to use SH-SY5Y cell line to screen the “Prestwick Chemical Library” using the compounds at the fixed concentration of 25 μM. This led to the identification of 4 molecules that significantly promote NDM29 expression (mitoxantrone dihydrochloride, perhexiline maleate, fendiline hydrochloride, alexidine dihydrochloride) and 6 that negatively modulate its transcription (tiabendazole, lisuride (S)(−), sulfathiazole, L-DOPA, pirenzepine dihydrochloride, zoxazolamine) (see Supplementary Fig. S2).

Among the drugs that promoted NMD29 expression we focused our attention on the anti-anginal drug perhexiline maleate (Fig. 1d), which was previously reported to act as modulator of induced resistance to chemotherapeutics as doxorubicin^20^,^21^.

**Figure 2:**
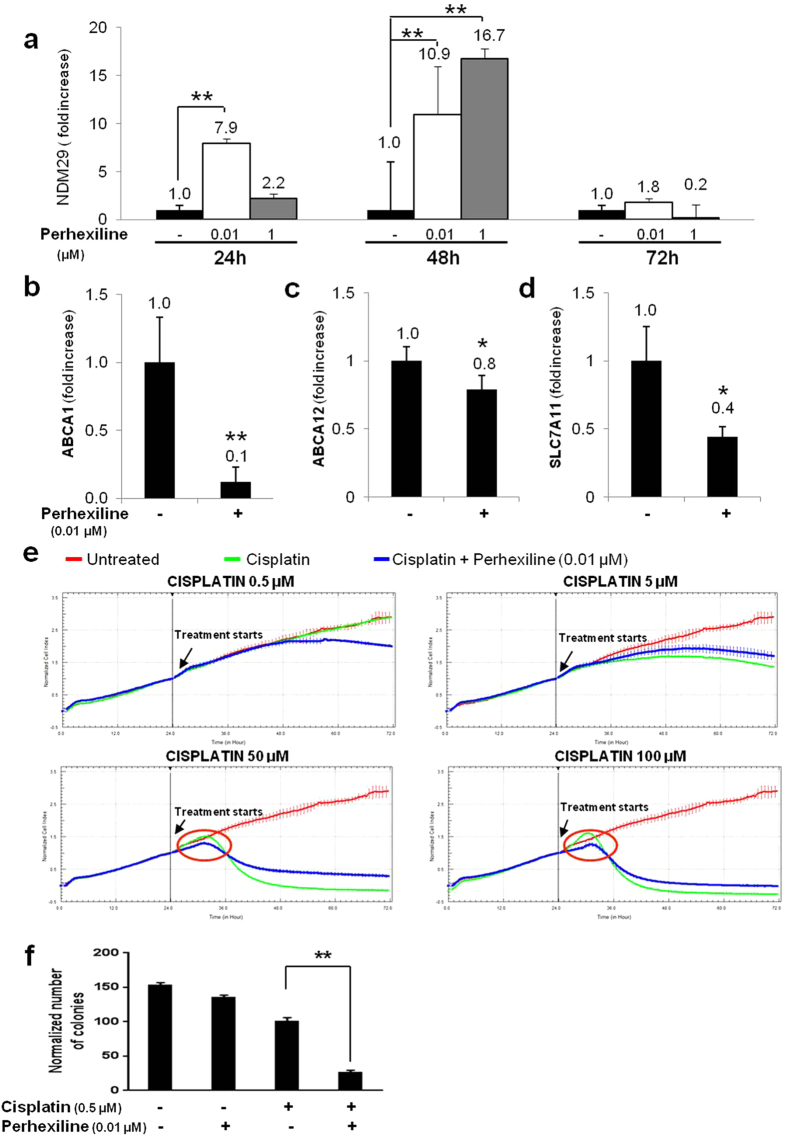
Perhexiline treatment increases the susceptibility of NB cells to antiblastic treatments. Real-time RT-PCR detection NDM29 mRNA amount in SH-SY5Y wt cells treated or untreated with perhexiline (0.01 and 1 μM) (24-48-72 h) (**a**). Values are mean ± SD. One star (*) indicates p < 0.05, while two stars (**) indicate p < 0.001, two-tailed Student’s test. Expression levels of ABCA1 ((**b**) p = 0.004), ABCA12 ((**c**) p = 0.0015) and SLC7A11 ((**d**) p = 0.0015) in SH-SY5Y wt cells treated with perhexiline (0.01 μM), measured by Real-time RT-PCR. One star (*) indicates p < 0.05, while two stars (**) indicate p < 0.001, two-tailed Student’s test. (**e**) Kinetics of cytotoxicity responses for cisplatin in SH-SY5Y wt cells, treated or not with perhexiline (0.01 μM), monitored by the RT-CES system. Cell Index (CI) was recorded every 30 minutes. Each trace at each concentration was an average of at least two replicates. Data are normalized to the time of compound addition of cell culture. (**f**) Capacity of SH-SY5Y wt to form colonies in methylcellulose in presence of different treatments (perhexiline 0.01 μM+ cisplatin 0.5 μM *vs.*cisplatin, p < 0.001). The data of two independent experiments are presented as the mean ± SD (n = 5 microscope fields for each treatment). The difference is significant as compared to cisplatin treatment: two stars (**) indicate p < 0.001, two-tailed Student’s test.

To monitor drug cytotoxicity in real-time and to establish the appropriate dosage for the selected small molecule, we used the xCELLigence system to measure SH-SY5Y cell proliferation in control and treated cells. As shown in Fig. 1e (see also Supplementary Fig. S3a), the concentrations used here were not toxic (0.01-0.1-1-25-50 μM). At 24, 48 and 72 h of treatment with 0.01 and 1 μM of perhexiline, NDM29 ncRNA expression level in SH-SY5Y cells (as resulting by Real-Time RT-PCR analysis) was progressively increased, reaching a peak after 48 hours of treatment (up to 10 and 16.7-fold with 0.01 μM and 1 μM, respectively), before declining after 72 hours (Fig. 2a).

Based on these results, we selected 48 hours after treatment initiation as the treatment endpoint for subsequent experiments.

### Perhexiline treatment increases the susceptibility of NB cells to antiblastic treatments

Next, we tested whether the up-regulation of endogenous NDM29 induced by perhexiline maleate might reproduce the biological effects observed upon its over-expression in NB cells, including the decrease of ABC transporters and the increased susceptibility to cisplatin.

We measured the expression level of ABCA1, ABCA12 and SLC7A11 in perhexiline (0.01 μM)-treated SH-SY5Y cells by Real-Time RT-PCR, and demonstrated that the enhanced expression of NDM29 was indeed associated with a reduced transcription of ABCA1, ABCA12 and SLC7A11 genes (p < 0.01 for ABCA1 and ABCA12, p < 0.05 for SLC7A11). These results suggest that perhexiline treatment might increase the efficacy of cisplatin (Fig. 2b–d). To test this hypothesis, we measured the viability of SH-SY5Y cells treated with several doses of cisplatin (0.5-5-50-100 μM) in the presence or absence of perhexiline (0.01 μM), and found that treated SH-SY5Y cells were more sensitive to cisplatin (see Supplementary Fig. S4a,b). In this experimental setting, at 24 hours of treatment, the sensitizing effect of perhexiline is clear at the highest concentration of cisplatin (100 μM) (cell viability was reduced of 15%, p = 0.0154) (see Supplementary Fig. S4a), whereas a prolonged treatment (for 48 hours) with this dose was toxic. For the two lower doses of cisplatin (0.5 and 5 μM) the highest effects were recorded after 48 hours (corresponding to peak of NDM29 over-expression) (p = 0.0473 and p < 0.001, respectively), suggesting a progressive, time-dependent accumulation of the drug molecules within the cell (see Supplementary Fig. S4b).

To assess whether perhexiline acts synergistically with cisplatin, we used the multiple drug effect analysis method of Chou and Talalay^22^,^23^, which quantitatively describes the interaction between two or more drugs^24^,^25^. At 24 h treatment, combination index (CI) values were consistently less than 1.0 only for the highest concentration of cisplatin (100 μM) (Combination Index  = 0.15231) indicating a strong synergism. At 48 h treatment, combination index values ranged from Combination Index  = 0.991 for the lowest dose (0.5 μM) of cisplatin, to Combination Index  = 0.59743 for cisplatin 5 μM, to Combination Index  = 0.73224 for cisplatin 50 μM, to Combination Index  = 0.80751 for the highest cisplatin dose (100 μM), indicating a synergistic interaction for the combination therapy with all the cisplatin doses tested.

These results, in agreement with previous experiments, indicate that the administration of perhexiline to SH-SY5Y cells increases their sensitivity toward co-administered cisplatin *in vitro*, potentiating its cytotoxic effects and acting in synergy.

These responses were confirmed by using the xCELLigence system to perform a kinetic analysis. We found that the combinations of perhexiline (0.01 μM) and cisplatin enhances the cytotoxic effects of the latter molecule (Fig. 2e and Supplementary Fig. S5a), as indicated by the observation of a decrease in cell survival in treated SH-SY5Y cells with 0.5 μM cisplatin, a concentration per se not effective (see Supplementary Fig. S5b). The same effect was observed at the highest doses, where perhexiline treatment leads to a more rapid cisplatin action (Fig. 2e).

A different treatment regime based on daily administration of perhexiline (0.01 μM) confirmed the increase of cisplatin efficacy in treated cells as well as its faster effects at the high dosages (see Supplementary Figs S5c,d and S6).

To verify that the sensitizing effect of perhexiline to cisplatin was due to NDM29, we used two permanently-transfected SH-SY5Y cell lines: SH-SY5Y Mock cells, transfected with pEGFPN1 plasmid, which express NDM29 at its basal level, and SH-SY5Y siNDM29 cells, in which NDM29 RNA level was decreased by 30% (p = 0.0012, Supplementary Fig. S7a).

As shown in Supplementary Fig. S7b, cisplatin was nontoxic in SH-SY5Y siNDM29 at the tested doses (0.05-0.5-5 μM). As expected the sensitizing effect of perhexiline to cisplatin was lost when NDM29 was knocked down in SH-SY5Y siNDM29 cells demonstrating again the crucial role played by NDM29 ncRNA in the modulation of cisplatin effects by perhexiline (Supplementary Fig. S8). Altogether, these results support the idea that co-administration of perhexiline maleate and cisplatin to inhibit NB cell proliferation might provide two beneficial effects: i) increased efficacy of the chemotherapic agent used at the standard dosage and ii) increased potency (i.e. reduction of the drug dose required to obtain antiproliferative effects) with a likely decrease of side effects.

### Co-administration of perhexiline maleate potentiates the efficacy of cisplatin to reduce the *in vitro* clonogenic potential of NB cells

To demonstrate that the decreased cell viability induced by perhexilline/cisplatin-treated cells is also associated to a reduction of NB cell tumorigenic potential, we investigated the effects of pharmacologically induced NDM29 RNA expression on the ability of SH-SY5Y cells to form colonies *in vitro*. Clonogenicity of SH-SY5Y, grown in methylcellulose and treated with cisplatin at the lowest concentration (0.5 μM), was tested in the presence or absence of perhexiline (0.01 μM).

For every tested condition, no differences were observed between untreated and perhexiline-treated cells in the morphology, size or number of colonies 12 days after seeding (see Supplementary Fig. S3b), confirming that this drug does not have innate anti-tumoral effects (Fig. 2f). On the contrary, the average colony number was reduced in the presence of cisplatin alone and almost abolished by the co-treatment of perhexiline with cisplatin (p < 0.001) (Fig. 2f). We also evaluated the capacity of forming colonies by SH-SY5Y siNDM29 cells, compared to Mock cells. Supplementary Fig. S9a,b show that SH-SY5Y siNDM29cells formed the same average number of colonies if treated with cisplatin alone or in combination with perhexiline, while for the control cells (Mock) the average colony number was reduced in the presence of perhexiline.

These results support the notion that combined treatment with perhexiline and cisplatin is able to decrease NB colony formation capacity by a mechanism NDM29-dependent, recapitulating the decreased clonogenicity of SKNBE2 permanently transfected with NDM29, previously reported^5^.

### Perhexiline, in combination with chemotherapy, reduces growth of NB *in vivo* and increases survival

Next, we investigated the ability of perhexiline to augment cisplatin toxicity on tumour cells *in vivo*, using a SK-N-BE(2) xenograft model. To evaluate whether this combination therapy might be useful to reduce the effective cisplatin doses (decreasing chemotherapy side effects), or to enhance the beneficial effects of a standard cisplatin dose, we performed 2 sets of experiments (protocol *a* and *b*: photographs of the dissected tumor masses are reported in Supplementary Figs S11 and S12).

**Figure 3:**
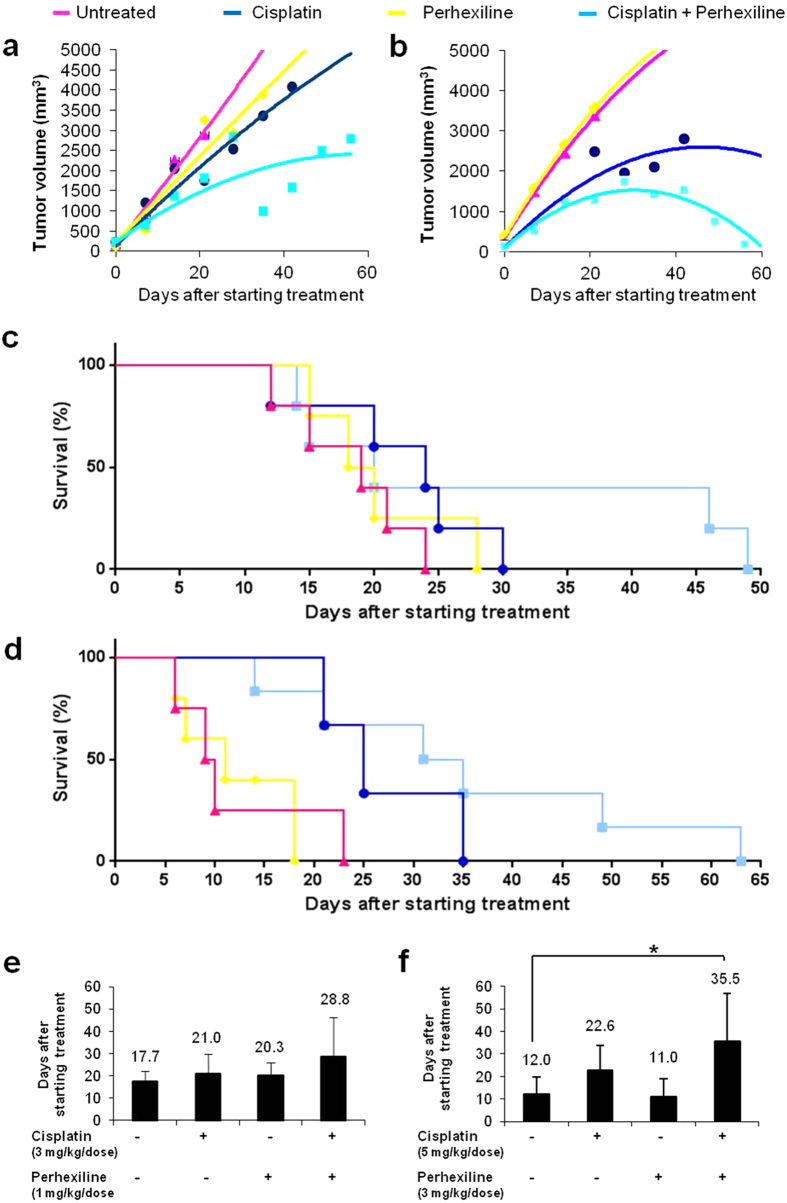
Perhexiline, in combination with chemotherapy, reduces growth of NB *in vivo* and increases survival. Kaplan-Meier log rank test for survival, showing the effect of perhexiline on cisplatin-induced reduction of SK-N-BE(2) xenograft growth in the protocol a (vehicle, Perhexiline 1 mg/Kg/dose and/or Cisplatin 3 mg/Kg/dose) (**a**) and in the protocol b (vehicle, Perhexiline 3 mg/Kg/dose and/or Cisplatin 5 mg/Kg/dose) (**b**). Treatment started when the tumour diameter 0.5 mm in size. NOD-SCID mice were randomized to receive the following treatments: vehicle (DMSO) daily by oral gavage (pink curves), perhexiline daily by oral gavage (yellow curves), cisplatin weekly by intraperitoneal injection (blue curves), or the combination (Combo) of perhexiline and cisplatin daily (cyan curves). Tumour volumes were measured weekly. Mice were sacrificed when the tumor size reached 2 cm^3^ or greater. Effect of perhexiline on percentaged survival of NOD-SCID mice in protocol a (vehicle, Perhexiline 1 mg/Kg/dose and/or Cisplatin 3 mg/Kg/dose) (**c**) and protocol b (vehicle, Perhexiline 3 mg/Kg/dose and/or Cisplatin 5 mg/Kg/dose) (**d**). Vehicle (DMSO): pink curves, Perhexiline: yellow curves, Cisplatin: blue curves, or the combination (Combo) of Perhexiline and Cisplatin: cyan curves. Mice were sacrificed when the tumor size reached 2 cm^3^ or greater. The histograms reported the days on average needed to reach tumour volume threshold in protocol a (vehicle, Perhexiline 1 mg/Kg/dose and/or Cisplatin 3 mg/Kg/dose) (**e**) and protocol b (vehicle, Perhexiline 3 mg/Kg/dose and/or Cisplatin 5 mg/Kg/dose) (**f**). Values are mean ± SD. The star (*) indicates p < 0.05, two-tailed Student’s test.

In the first set of experiments (protocol *a*), which used low cisplatin dosage, we found that the control groups (mice treated with DMSO or 1 mg/kg/dose e.g. perhexiline alone) rapidly developed tumours with a constant linear tumour growth (Fig. 3a), and showed no significant difference in survival (means of overall progression-free survival were 17.7 and 20.3 days respectively; p = 0.4374), confirming that perhexiline *per se* does not have any antitumor effects (Fig. 3c).

Consistent with the low dose used, the cisplatin-treated group (3 mg/kg/dose of cisplatin) showed only a slightly decrease in tumour growth rate (Fig. 3a), with progression-free survival similar to that of control groups (mean overall progression-free survivals was 20.3 for cisplatin-treated group *vs.* 17.7 and 21 days for mice treated with DMSO (p = 0.406) or perhexiline (p = 0.8855), respectively; Fig. 3c). However, the co-administration of perhexiline and cisplatin yielded a clear enhancement of antitumor effects, resulting in a significantly improved progression-free survival as compared with mice treated with DMSO (p = 0.1589), perhexiline (p = 0.3447) or cisplatin (p = 0.3534) alone (mean overall progression-free survivals were 28.8 days *vs.*17.7, 20.3 and 21 days respectively; Fig. 3c).

In a second set of experiments, we studied whether the combination of cisplatin and perhexiline might induce stronger effects than each drug alone, when high doses of both are used (3 mg/kg/dose of perhexiline, 5 mg/kg/dose of cisplatin). Consistent with the results reported above, mice treated with cisplatin and perhexiline had significantly lower tumour growth rates compared to controls: indeed, perhexiline plus cisplatin led to a significant reduction of tumour growth compared with the other groups (cisplatin, perhexiline or DMSO; Fig. 3b). As shown in Fig. 3d, mean overall progression-free survival was 12 days in the control group versus 11, 22.6, and 35.5 days in groups treated with either perhexiline (p = 0.8124), cisplatin (p = 0.0805), or perhexiline plus cisplatin (p = 0.0414), respectively. Combination therapy also increased progression-free survival in a statistically significant manner compared to either DMSO or perhexiline treatment (p = 0.0414 and p = 0.0168, respectively), although the comparison with cisplatin alone did not reach statistically significance (p = 0.1746).

Taken together these findings indicate that perhexiline does not have antitumor effects as a single agent, but it is able to enhance the antitumor effects of cisplatin, consistent with the *in vitro* data described above.

### Perhexiline enhances cisplatin cytotoxicity *in vivo*

To characterize the effects of the pharmacological treatments described above on NB tumours, histological and molecular analyses were performed on tumors explanted from treated and untreated mice.

**Figure 4:**
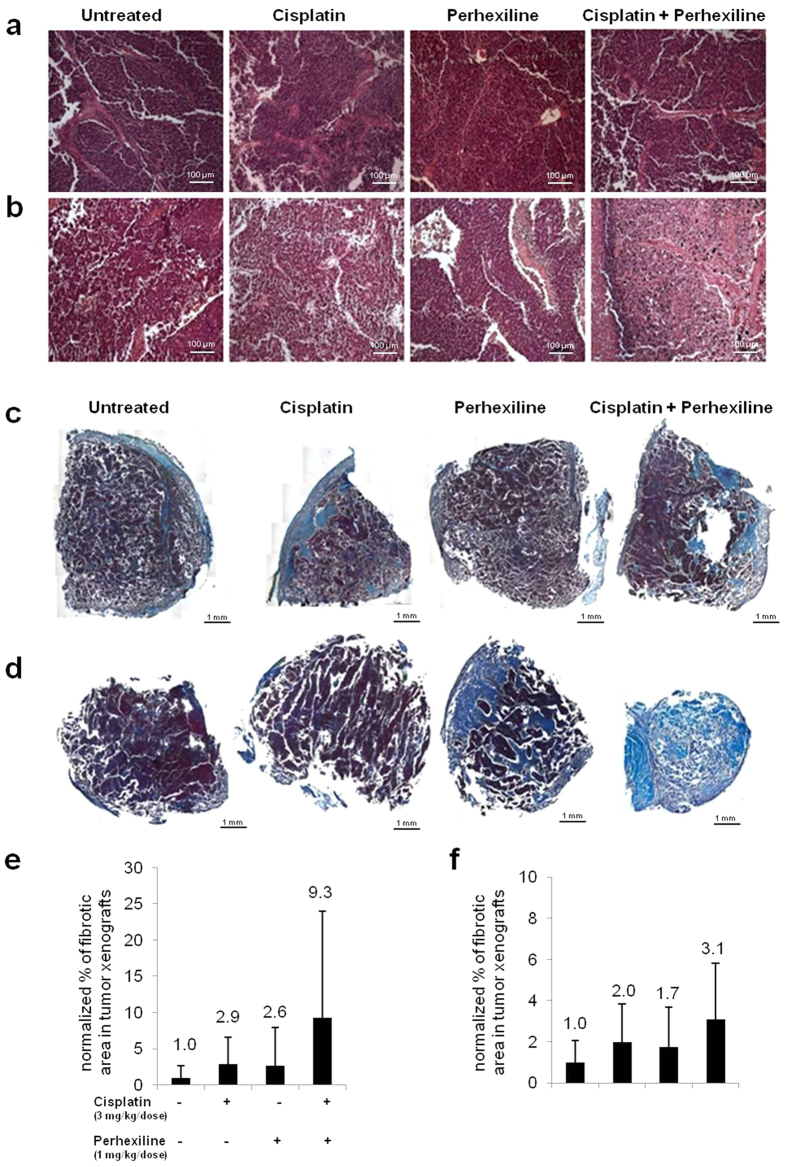
Perhexiline increases *in vivo* cisplatin cytotoxicity in a NB mouse model. Representative light microscopy images of haematoxylin/eosin stained sections of subcutaneous tumours in treated and untreated mice, in protocol *a* (vehicle, Perhexiline 1 mg/Kg/dose and/or Cisplatin 3 mg/Kg/dose) (**a**) and *b* (vehicle, Perhexiline 3 mg/Kg/dose and/or Cisplatin 5 mg/Kg/dose) (**b**). Scale bar: 100 μm. Representative images of treated and untreated tumours, stained by Mallory’s trichrome (magnification 20×), from protocols *a* (vehicle, Perhexiline 1 mg/Kg/dose and/or Cisplatin 3 mg/Kg/dose) (**c**) and *b* (vehicle, Perhexiline 1 mg/Kg/dose and/or Cisplatin 3 mg/Kg/dose) (**d**). Scale bar: 1 mm. Mean fibrotic area in tumour specimen from each mouse from protocols *a* (**e**) and *b* (**f**) was reported as histograms.

An analysis of morphology and size of the cells within tumour nodules from control or perhexiline and/or cisplatin treated mice did not reveal differences: all tumours were comprised of sheets and cords of small undifferentiated cells typical of NB (Fig. 4a,b).

To evaluate a possible correlation between the reduction of tumour growth induced by the combination therapy and cisplatin cytotoxicity, we measured the fibrotic area (index of tumour necrosis) in tumour specimens from each mouse (Fig. 4c,d). In both *in vivo* sets of experiments, tumors from mice treated with both drugs showed larger mean fibrotic area compared to all other groups (Fig. 4e,f), although not in a statistically significant manner. Moreover, in both *in vivo* sets of experiments, annexin V measurements (see Supplementary Fig. S10) revealed that perhexiline *in vivo* treatment did not induce apoptosis or necrosis (confirming that it is not an antitumoral drug), whereas cisplatin treatment induced apoptosis at higher rate than occurred in the control group, but necrosis was not observed. In mice treated with combination therapy, an increase in apoptosis was observed, similar to cisplatin-treated mice, accompanied by a not significant decrease of necrosis (more than cisplatin), probably due to the replacement of the necrotic area with fibrosis.

### Perhexiline favours NB cell transition to differentiated phenotype *in vivo*

Considering that NDM29 over-expression promotes differentiation also within the tumour initiating cell (TIC) fraction, we hypothesized that the combination therapy might render the elusive cancer stem cells more vulnerable to cisplatin.

**Figure 5:**
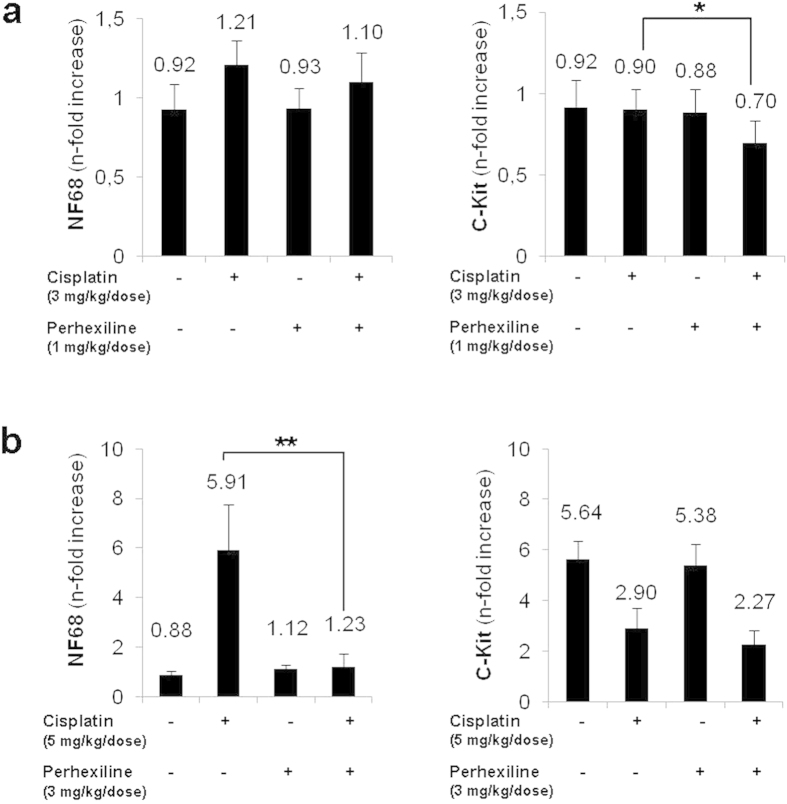
Perhexiline favours NB cell transition to differentiated phenotype *in vivo*. Real-time RT-PCR quantification of differentiation marker (NF68) and stemness marker (C-Kit), in the tumour nodules. Protocol *a* (vehicle, Perhexiline 1 mg/Kg/dose and/or Cisplatin 3 mg/Kg/dose) (**a**), protocol *b* (vehicle, Perhexiline 3 mg/Kg/dose and/or Cisplatin 5 mg/Kg/dose) (**b**). One star (*) indicates p < 0.05, while two stars (**) indicate p < 0.001, two-tailed Student’s test.

To verify this hypothesis, we measured by Real-time RT-PCR the expression level of Neurofilament 68, a marker of NB cell differentiation, (NF68, RefSeq NM_006158.4) and of C-Kit, associated with self-renewal capacity (C-Kit, RefSeq NM_000222), in the nodules derived from treated and untreated mice. Figure 5a,b show that cisplatin treatment alone was sufficient to trigger increased expression of NF68 as well as a decrease in C-Kit expression in tumors of mice treated with a high dose of the drug. In the low cisplatin dose group however, NF68 upregulation was associated with a significant decreased expression of C-Kit only in nodules derived from mice treated with both cisplatin and perhexiline (p < 0.05 in protocol *a*).

These results suggest that while cisplatin may induce differentiation of actively cycling tumor cells, the combination with perhexiline leads to a significant reduction of the TIC fraction.

Altogether, these data indicate that perhexiline treatment enhances the effects of cisplatin, decreasing the growth rate and stem-like phenotype of human tumour cells and increasing differentiation, consistent with what has been previously reported in NDM29 over-expression experiments.

## Discussion

NB is characterized by high biological diversity evident by the presence of heterogeneous cellular subtypes within tumors (N, I, S cells), and of cancer stem cell population, responsible for recurrence and drug resistance^26^,^27^,^28^,^29^,^30^. Although advances in surgical approaches, radiotherapy, and chemotherapy are contributing to incremental improvements in prognosis and survival of paediatric cancer patients, not much progress, especially concerning the rates of metastatization in advanced patients, recurrence, and long term survival, has been made in the therapy of neuroblastoma^1^.

Moreover, there is an urgent need for the clinical development of safe and nontoxic cytoprotective agents for the adequate management of the adverse effects of cancer chemotherapy^31^.

We recently demonstrated that stable over-expression of NDM29 ncRNA induces NB cell differentiation into a non-malignant neuron-like phenotype^5^ characterized by typical biochemical and electrophysiological traits^5^,^8^,^9^,^11^. In addition, we reported that NDM29-induced differentiation affects the activity of tumour initiating/cancer stem cells (TICs)^5^, that represents an ineludible drug target to prevent tumour relapses. This observation suggests that the pharmacological induction of endogenous NDM29 synthesis could represent a starting point for a novel therapeutic approach to be used with traditional cytotoxic therapy, with the aim of rendering the elusive cancer stem cells more susceptible to anticancer drugs. We found that this differentiation process is accompanied by the down-regulation of ABC transporters, whose expression is known to enhance chemoresistance, thus increasing the sensitivity of cancer cells to cisplatin. In this context, the identification of compounds that might pharmacologically induce the expression of NDM29 ncRNA is a logical next step. It is conceivable that the co-administration of NDM29 inducers with cisplatin might reduce the required dosage and/or increase the chemotherapeutic efficacy in the treatment of NB nodules.

Thus, the main aim of this study was to identify possible modulators of NDM29 expression and to evaluate their *in vitro* and *in vivo* antitumor activity in combination with cisplatin.

To overcome the limitations of screening large libraries, we choose to study a limited number of molecules already available as drugs in humans, in accordance to the “selective optimization of side activities” (SOSA) approach^12^. For this reason, we used the Prestwick Chemical Library, which consists of 1,120 compounds structurally and therapeutically very different but all of them having known safety and bioavailability in humans.

The deep screen of this library allowed us to identify a small molecule, perhexiline maleate, able to induce NDM29 expression. This drug was first introduced in the 1970 s as an effective anti-anginal agent, improving exercise tolerance and increasing the workload needed to induce ischaemia^32^. Perhexiline is thought to act by inhibiting mitochondrial carnitine palmitoyltransferase-1, shifting myocardial metabolism from fatty acid to glucose utilisation, which increases ATP production without affecting O_2_ consumption, consequently improving myocardial efficiency^33^. Although perhexiline maleate has been reported to be a calcium antagonist it was demonstrated that this drug concurrently administrated with doxorubicin also enhanced doxorubicin inhibition of proliferation of the anthracycline-resistant P388 cell sub-line in a reversible way^20^,^21^. Recently, it has been reported that perhexiline is also a HER3 ablation modulator that inhibits breast cancer cell proliferation *in vitro* and *in vivo,* having a synergistic inhibitory effect with lapatinib on tumor growth^34^. Nonetheless, the anti-cancer mechanism of perhexiline remains still unclear.

In this work we demonstrated that perhexiline increases NDM29 expression and recapitulates the biological effects observed in NB cells genetically over-expressing NDM29^5^, conferring susceptibility to the effects of a cytotoxic drug commonly used in NB therapy, cisplatin. Conversely, no effects on NB cell viability or proliferation were observed by the treatment with perhexilline alone. In addition, perhexiline increases *in vivo* cytotoxicity of cisplatin in a NB mouse model: NOD-SCID mice receiving cisplatin plus perhexiline had significantly better progression-free survival rates and lower tumour growth rates as compared with the control groups. The enhanced cisplatin effect due to perhexiline co-administration is probably dependent on increased fibrotic areas and apoptosis within tumour nodules that is accompanied by a marked, but not statistically significant, decrease of necrosis (more than cisplatin). This observation is likely due to the substitution of necrotic area by fibrotic tissue, as previously demonstrated^35^, and to the documented inverse correlation between cisplatin-induced apoptosis and necrosis: apoptosis induced by cisplatin seems to precede necrosis when the apoptotic machinery is operative; on the contrary, when the apoptosis program is defective, necrotic cell death takes place as an alternative pathway leading to cell demise^36^. Moreover, the combination therapy significantly reduces the TIC fraction of the tumour cell population, as demonstrated by the concomitant decrease of stemness marker C-Kit and increase of NB differentiation marker NF68.

In the future, possible synergistic effects induced by the combined treatment of perhexiline with other frequently administered chemotherapeutic agents should be explored.

In summary, the *in vitro* and *in vivo* results raise the possibility of using this type of combination therapy to kill non-stem cancer cells and simultaneously to promote differentiation of “stem-like” cells to more susceptible tumour cells to the action of anticancer drugs.

## Methods

### Cell cultures

SKNBE2 wt, Mock and S1 cells were maintained on RPMI 1640 medium (Sigma–Aldrich, Milan, Italy), 10% FBS (GIBCO, S.Giuliano Milanese, Milan, Italy), L-glutamine (2 mM; EuroClone, Devon, UK), penicillin–streptomycin (100 U/ml/100 ug/ml; EuroClone) (standard medium). SH-SY5Y wt cells were maintained on Dulbecco’s modified Eagles medium (DMEM) (Sigma–Aldrich), 10% FBS (GIBCO), L-glutamine (2 mM; EuroClone), and penicillin–streptomycin (100 U/ml/100 ug/ml; EuroClone). SH-SY5Y wt cells were stably transfected using polyethylenimine (PEI; Sigma P3143) with pEGFPN1 as control (hereafter referred to as Mock) or pEGFPN1-siNDM29 (hereafter referred to as siNDM29). G418 (Geneticin; Invitrogen) was used in culture medium as mean of selection up to 1000 μg/ml, until resistant clones were identified. After selection, the clones were preserved in 200 μg/ml G-418 in standard culture conditions.

### Quantitative Real-Time RT-PCR analysis

Total RNAs from samples were extracted using TRIzol reagent (Invitrogen, Carlsbad, CA, USA) according to the manufacturer’s protocol and subjected to reverse transcription by Transcriptor First Strand cDNA Synthesis Kit (Roche, Germany), with random hexamer as primers, following manufacturer’s instructions. The total RNA from samples was measured by real-time quantitative RT-PCR using PE ABI PRISM@ 7700 Sequence Detection System (Perkin Elmer Corp./Applied Biosystems, Foster City, CA) and Sybr Green method following manufacturer’s instructions. The sequences of forward and reverse primers were reported in Supplementary Table S1. No-template control tubes (NTC), containing water instead of template mRNA, were also run under the same conditions for each gene. Relative transcript levels were determined from the relative standard curve constructed from stock cDNA dilutions, and divided by the target quantity of the calibrator following manufacturer’s instructions.

### cDNA synthesis and Real Time-PCR array

The cDNA for each RNA sample was obtained using RT^2^ First Strand kit (SABiosciences Corporation, QIAGEN Company, Frederick, MD) according to the manufacturer’s instructions. The cDNA was loaded into each well of the RT^2^ Profiler PCR Array (SABiosciences Corporation, Cat. No. PAHS-070 SABiosciences). PCR array experiments were performed on an Eppendorf Realplex^4^ EP gradient S Mastercycler^®^. The PCR array data were analyzed by PCR Array Data Analysis web-based software (SABiosciences Corporation).

### MTT assay

The effect of antitumoral drugs on neuroblastoma cell survival was evaluated using the MTT assay. Approximately 24 hours after plating, cells were exposed to the tested drugs for 48 hours at 37 °C. At the end of the treatment, the medium was removed and cells were incubated for 1 hr in MTT solution (2 mg/ml in PBS) (Sigma Aldrich, Milan, Italy). After removing MTT, 500 μl of absolute ethanol were added to each well to dissolve the formazan crystals, and absorbance for each well was determined using a spectrophotometer at 570 nm with a reference filter at 670 nm. Cytotoxicity was expressed as the percentage of cells surviving in relation to untreated cells.

### Determination of Combination Index (CI) values

Drug-induced cytotoxic synergy was analyzed using the median-effect method^22^,^23^, and it was expressed as the combination index (CI), used to assess the nature of drug-drug interactions that can be additive (CI = 1), antagonistic (CI > 1), or synergistic (CI < 1) for various drug-drug concentrations^37^,^38^. CI values were calculated using CompuSyn software (ComboSyn Inc., Paramus, NJ, USA), following the method by Chou *et al.* Based on CI, synergy is further refined as moderate synergism (combination index = 0.7–0.9), synergism (combination index = 0.3–0.7), strong synergism (combination index = 0.1–0.3), and very strong synergism (combination index < 0.1)^22^.

### Cytotoxicity assays using xCELLigence system

Cell proliferation and cytotoxicity were assessed by xCELLigence RTCA MP System (Roche, Germany) that monitors cellular events in real time by measuring electrical impedance across interdigitated gold micro-electrodes integrated on the bottom of tissue culture plates. Cell-sensor impedance is expressed as an arbitrary unit called Cell Index (CI). Briefly, SH-SY5Y wt, Mock and siNDM29 cells (7*10^4^ cells per well) were seeded into 100 μL of standard medium in 96X microtiter plates (E-Plate, Roche, Germany), and, approximately 24 hours later, compounds were added to the wells.

Perhexiline was dissolved in DMSO (0.1%). DMSO controls were performed along with the drugs experiments to ensure that any observed cytotoxic effects were due to drugs and not to DMSO.

Cell proliferation was monitored for 72 hours or more. Cell attachment, spreading and proliferation were monitored every 30 minutes using the xCELLigence system to produce time-dependent cell response dynamic curves. A range of perhexiline (0.01–50 μM) and cisplatin (0.5–100 μM) concentrations were tested separately or in combination on SH-SY5Y cells. Each concentration was repeated at minimum in duplicate. Analyses were performed by RTCA Software 1.2 of the xCELLigence system.

### Methylcellulose colony formation assay

Clonogenic assays were performed using a methylcellulose medium consisting of DMEM with 0.4% methylcellulose (Methocult H4100, StemCell Technologies, Vancouver, BC, Canada), 10% fetal bovine serum, 100 U/mL penicillin/streptomycin, and 2 mM L-glutamine. SH-SY5Y wt or Mock or siNDM29 cells were plated at a density of 500 cells in methylcellulose medium in humidified 6-well plates. Colonies were counted 12 days after plating. Images were captured at 20× or 4× magnification on an EVOS fl digital inverted microscope (Advanced Microscopy Group, WA, USA). Data were obtained from two independent assays performed in duplicate. Each data represents an average of five microscope fields for each treatment.

### Screening assay

The Prestwick Chemical Library (Illkirch, France) which consists of 1,120 drugs and bioactive natural compounds approved by the FDA was screened on SH-SY5Y cells stably expressing luciferase. 45*10^3^ cells were seeded into 96-well plates (Greiner Bio-one, Courtaboeuf Cedex, France), and transfected using PEI (polyethylenimine) (SIGMA P3143, Sigma- Aldrich) in accordance with the manufacturer’s instructions (100 ng plasmid DNA per well, complexed with PEI). We used pSHAG magic2-Promoter NDM29, in which NDM29 promoter is fused to a luciferase-specific silencer hairpin. An efficient hairpin transcription driven by NDM29 promoter was detectable as a significant decrease of luciferase activity; on the contrary, in all the cases in which the treatment was exerted an inhibitory effect on the promoter of interest the correspondent luciferase-specific silencing hairpin was transcribed with a decreased efficiency resulting in a more active luciferase emission quantitatively detectable. As controls, we used pSHAG magic2-No promoter. An EGFP expression vector (pEGFP-N1) was also co-transfected for determination of transfection efficiency. After the transfection, the medium was exchanged for complete medium containing drugs (25 μM in 0.1% DMSO) or 0.1% DMSO as control. After 36 hours, the luciferase-based promoter activity assay was performed by firefly luciferase activity determination with firefly luciferase/luciferin bioluminescent system (Biosynth AG, Staad, Switzerland) according to the manufacturer’s protocol. Luminescence readings were taken on the Tecan GENios Pro microplate reader (1 second integration time/sample) (Tecan, Männedorf, Switzerland). Luminescence in each treated well was compared to the mean signal obtain in untreated wells. Positive hits were designated for any compounds with luminescent signal over three standard deviations when compared to untreated control wells. Luminescent signal is normalized to amounts of GFP, measured by the microplate reader, for normalization of transfection efficiency.

### Mice

Homozygous NOD-SCID (NOD.CB17-Prkdcscid) mice were purchased from the Jackson Laboratory (Bar Harbor, MA, USA). Mice were used between 5 and 8 weeks of age. All animals were bred and maintained at the institution’s animal facility of the National Institute for Cancer Research (Genoa, Italy).

All experimental procedures were carried out in accordance with the guidelines of the European Community for the use and care of live animals, approved by the Italian Ministry of Health as well as by the Ethics Committee of the Animal Facility of the National Institute for Cancer Research (IST, Genova, Italy) (with protocol DGSAF 0001448-A). Efforts were made to minimize animal stress/discomfort.

### *In vivo* tumorigenicity assay

A cell suspension of SKNBE2 wt cells (5*10^6^ cells) in PBS was subcutaneously injected into NOD/SCID mice. Mice were divided in several groups and 2 protocols were followed.

In protocol *a*, 21 mice were divided in 4 groups: control vehicle group: DMSO (n = 6 mice); cisplatin (3 mg/kg/dose) treated group (n = 6); perhexiline (1 mg/kg/dose) treated group (n = 4) and; perhexiline (1 mg/kg/dose) and cisplatin (3 mg/kg/dose) treated group (n = 5).

In protocol *b*, 20 mice were divided in 4 groups: control vehicle group: DMSO (n = 4 mice); perhexiline (3 mg/kg/dose) treated group (n = 5); cisplatin (5 mg/kg/dose) treated group (n = 5); perhexiline (3 mg/kg/dose) and cisplatin (5 mg/kg/dose) treated group (n = 6).

In both protocols, cisplatin was administered i.p. weekly, while perhexiline as well as DMSO were administered intragastrically, once a day/5 days a week by gavage, in which a feeding needle was introduced into the esophagus and the drugs were delivered directly into the stomach.

Treatments began when neoplasia reached a threshold diameter of 5 mm. Mice were observed weekly for the appearance of tumors at injection sites. Tumor size was measured every week with calipers in all groups caliper and tumor volume was calculated by the formula length^2^ × width × π/6. Imaging of each mouse was collected at each considered time point. Mice injected *s.c.* were sacrificed when the tumor size reached 2,200 mm^3^ or greater.

To calculate the tumor development function by the polynomial interpolation, we used the average of the daily measures when each mouse had the tumor mass. For each group we also analyzed the averaged survival time after starting treatment.

### Cell cultures derived from tumors

Portions of each considered tumor were washed in PBS and digested with 12.5 U/ml type I Collagenase (Biochrom AG, Berlin, Germany) and 12 U/ml Dispase (Roche, Germany) in PBS for 20 min at 37 °C. Freshly-isolated cells were used for both flow cytometric analysis and *in vitro* expansion in standard medium.

### Histological analysis

For histological examination, tumors derived from each experimental group were surgically removed and fixed in 4% neutral-buffered formalin, dehydrated and embedded in paraffin using standard histologic techniques. Serial 5 μm sections were cut and stained with hematoxylin and eosin (H/E) to examine morphologic features, and Mallory’s trichrome (MT) for detection of collagen fibers. Images were captured by transmitted light microscopy using a Zeiss Axiovert 200 M microscope equipped with a Zeiss Axio-Cam MRc color chilled 3CCD camera (Zeiss, Wetzlar, Germany). The H/E-stained slides were observed under the same transmitted light microscope. The collagen volume fraction per tumour was determined using Image J software. The percentage of fibrosis was obtained by dividing the total area of collagen by the total area of the section.

### Apoptosis analysis

Apoptosis was analyzed by flowcytometry, using Annexin V (Annexin V-FITC Apoptosis Detection Kit I; BD Biosciences, Oxford, UK) according to the manufacturer’s instructions. The samples were analyzed using Cyan ADP cytofluorimeter (Beckman-Coulter, Brea, CA, USA). For each sample, 20,000 events were acquired. The data was analyzed using Summit 4.3.1 software (Dakocytomation, Ely, Cambridge, UK).

### Statistical analysis

Results are expressed as mean ± Standard Deviation. Statistical significance of observed differences among different experimental groups was calculated using a two-tailed unpaired Student’s *t* test. A P value of less than 0.05 was considered to be statistically significant. In the figures, * and ** indicate statistical significance at p < 0.05 and 0.01, respectively. For survival studies, Kaplan-Meier curves were plotted and compared using the log-rank test. The statistical calculations were performed with GraphPad Prism 6.0 for Windows (GraphPad Software, La Jolla, CA, USA).

## Additional Information

**How to cite this article**: Vella, S. *et al.* Perhexiline maleate enhances antitumor efficacy of cisplatin in neuroblastoma by inducing over-expression of NDM29 ncRNA. *Sci. Rep.* **5**, 18144; doi: 10.1038/srep18144 (2015).

## Supplementary Material

Supplementary Information
