# Supplementary material for: Perhexiline maleate enhances antitumor efficacy of cisplatin in neuroblastoma by inducing over-expression of NDM29 ncRNA
Source: Sci Rep. 2015 Dec 17;5:18144. doi: 10.1038/srep18144 (PMC4682181; doi:10.1038/srep18144)
Supplement: Supplementary Information [file srep18144-s1.pdf]

Perhexiline maleate enhances antitumor efficacy of cisplatin in neuroblastoma by inducing over-expression of NDM29 ncRNA

Serena Vella, Ilaria Penna, Luca Longo, Giulia Pioggia, Patrizia Garbati, Tullio Florio, Fabio Rossi, Aldo Pagano

Supplementary Figure S1. Results of Human drug transporter PCR Array in S1 cells, compared to Mock cells.

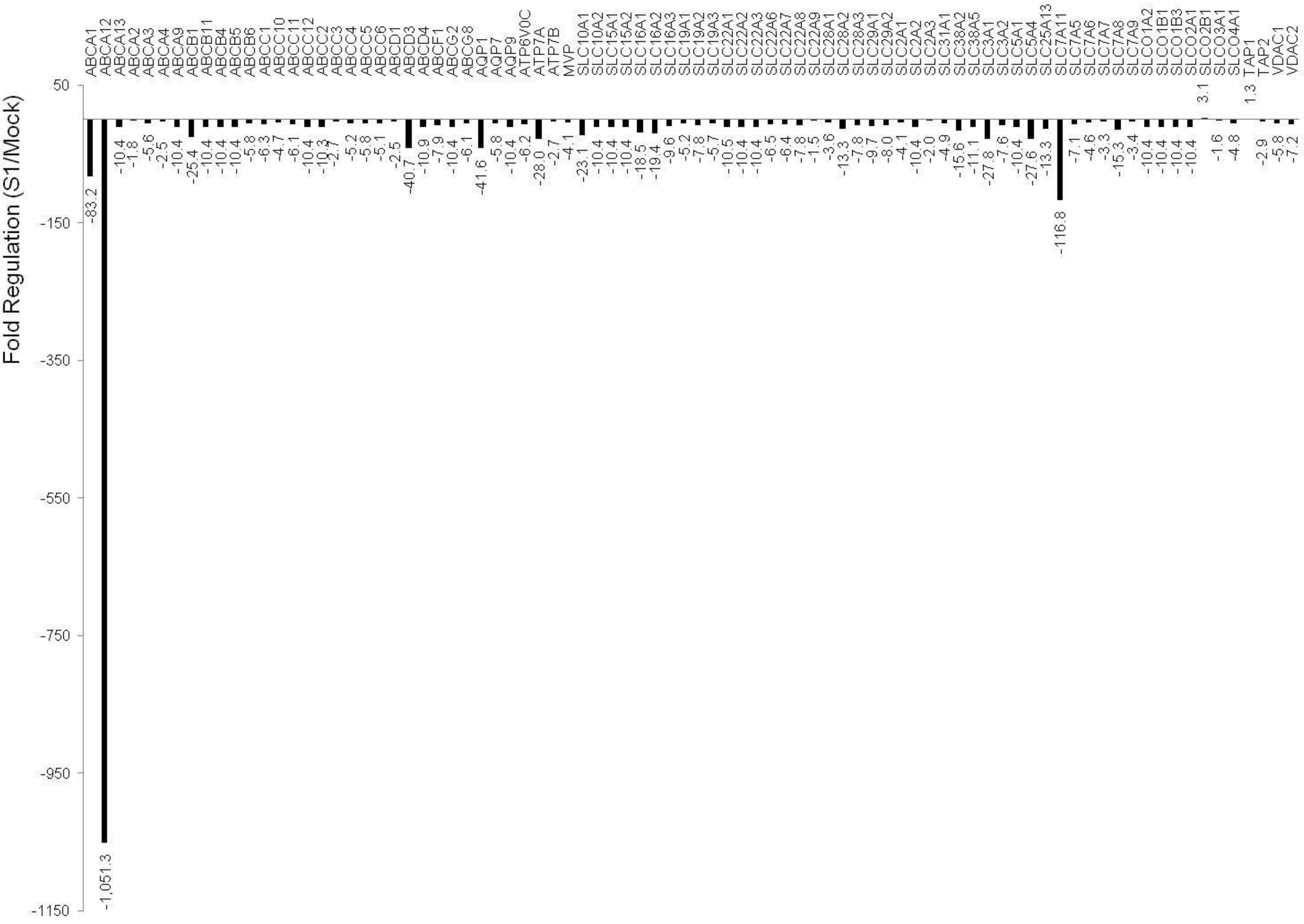

Supplementary Figure S2. Results of the primary screening. Results are reported as luciferase emission of treated *vs.* untreated cells. Luminescent signal is normalized to amounts of GPF of cells, for normalization of transfection efficiency.

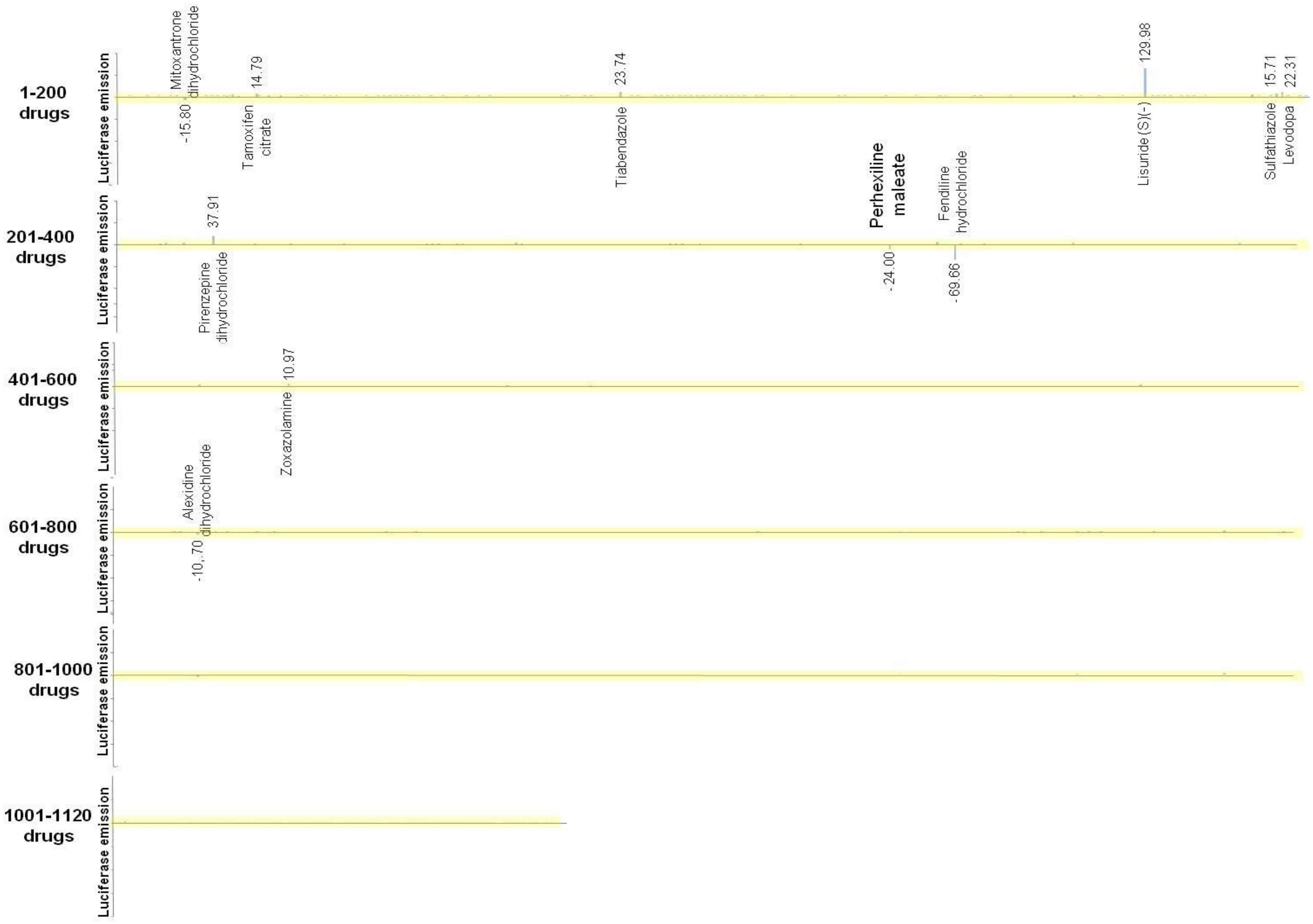

Supplementary Figure S3. **a**) Phase contrast microscopy of SH-SY5Y wt cells cultured in the absence or presence of perhexiline (0.01 and 1 µM). Cells were monitored at various times up to 72 hr by phase contrast microscopy at 20X. Scale bar: 200 µm.
b) Two representative pictures of colonies at day 12 after seeding for each experimental conditions (perhexiline 0.01 µM and/or cisplatin 0.5 µM). Magnification 20X. Scale bar: 200 µm

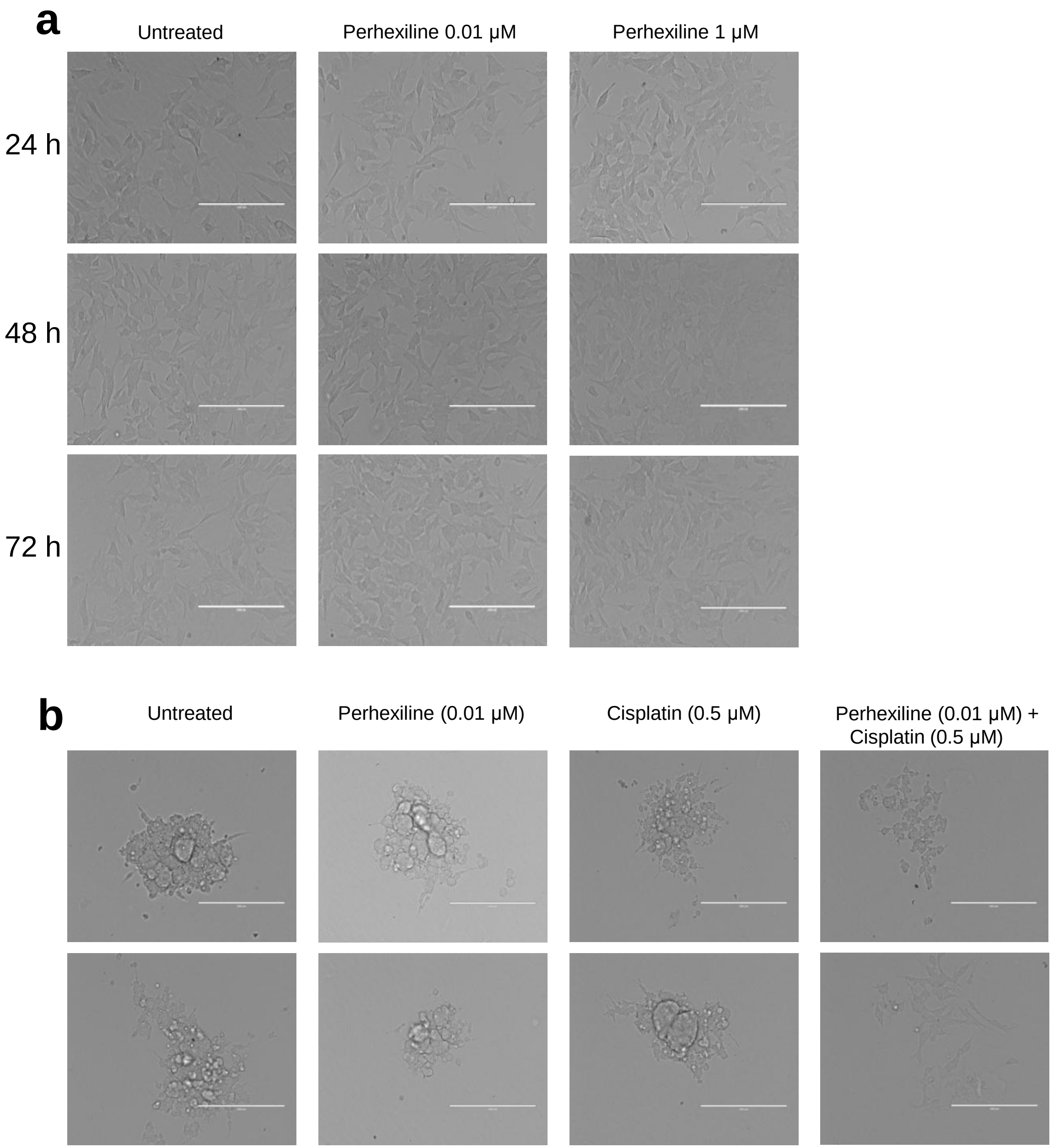

Supplementary Figure S4. Effects of perhexiline (left panel) (0.01 μM) or cisplatin (middle panel) or the combination cisplatin/ perhexiline (right panel) on SH-SY5Y cell viability (MTT assay) after 24 (**a**) and 48 (**b**) hours of treatment. Perhexiline 0.01 μM; Cisplatin 0.5-5-50-100 μM. One star (*) indicates p<0.05, while two stars (**) indicate p<0.001.

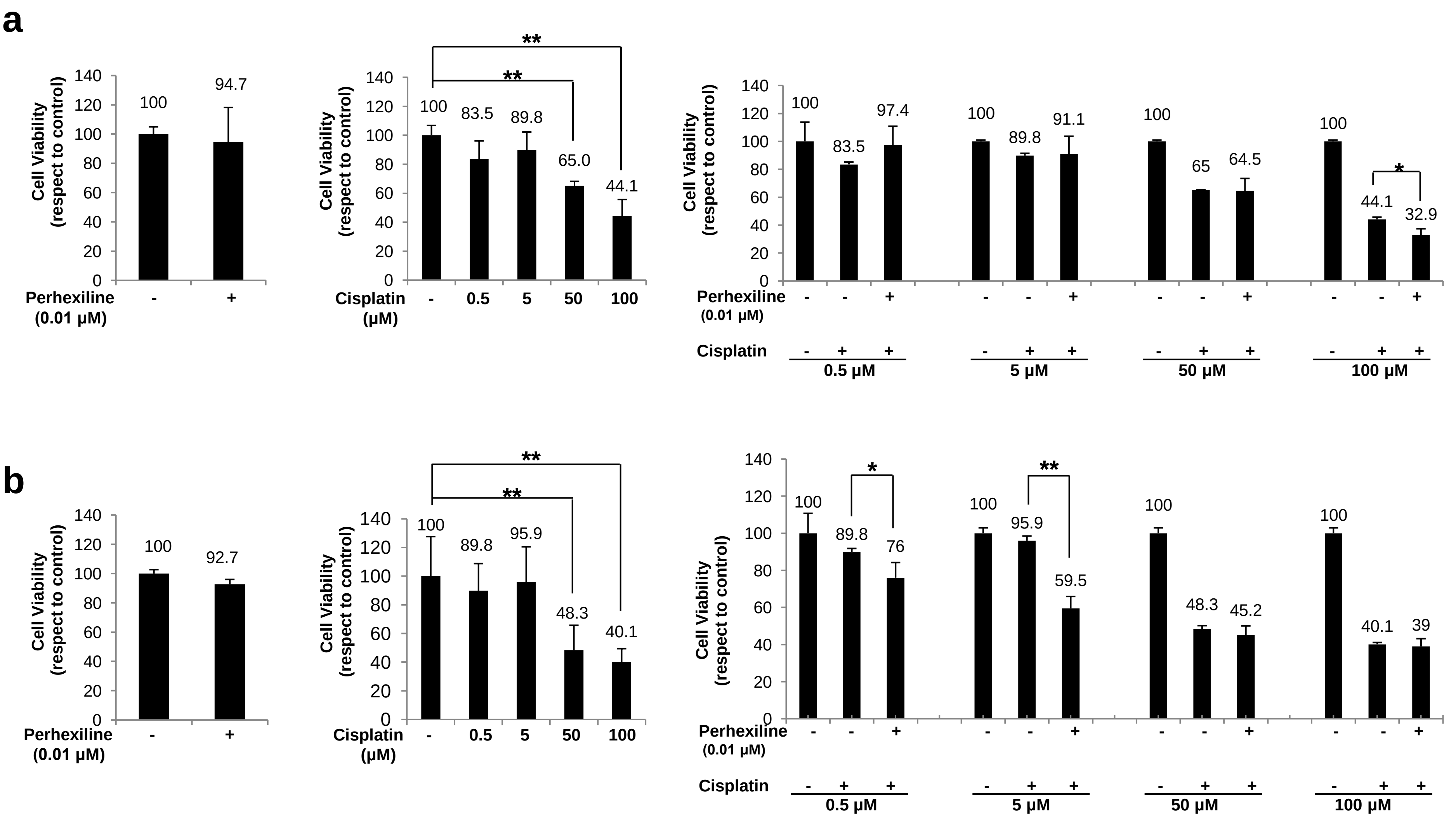

Supplementary Figure S5. Kinetics of cytotoxicity responses for cisplatin (0.5-5-50-100 μM) in SH-SY5Y wt cells, daily treated or not with perhexiline (0.01 μM), monitored by the RT-CES system. Different compound concentrations are indicated by different colours. CI was recorded every 30 minutes. Each trace at each concentration was an average of at least two replicates. Data are normalized to the time of compound addition of cell culture.

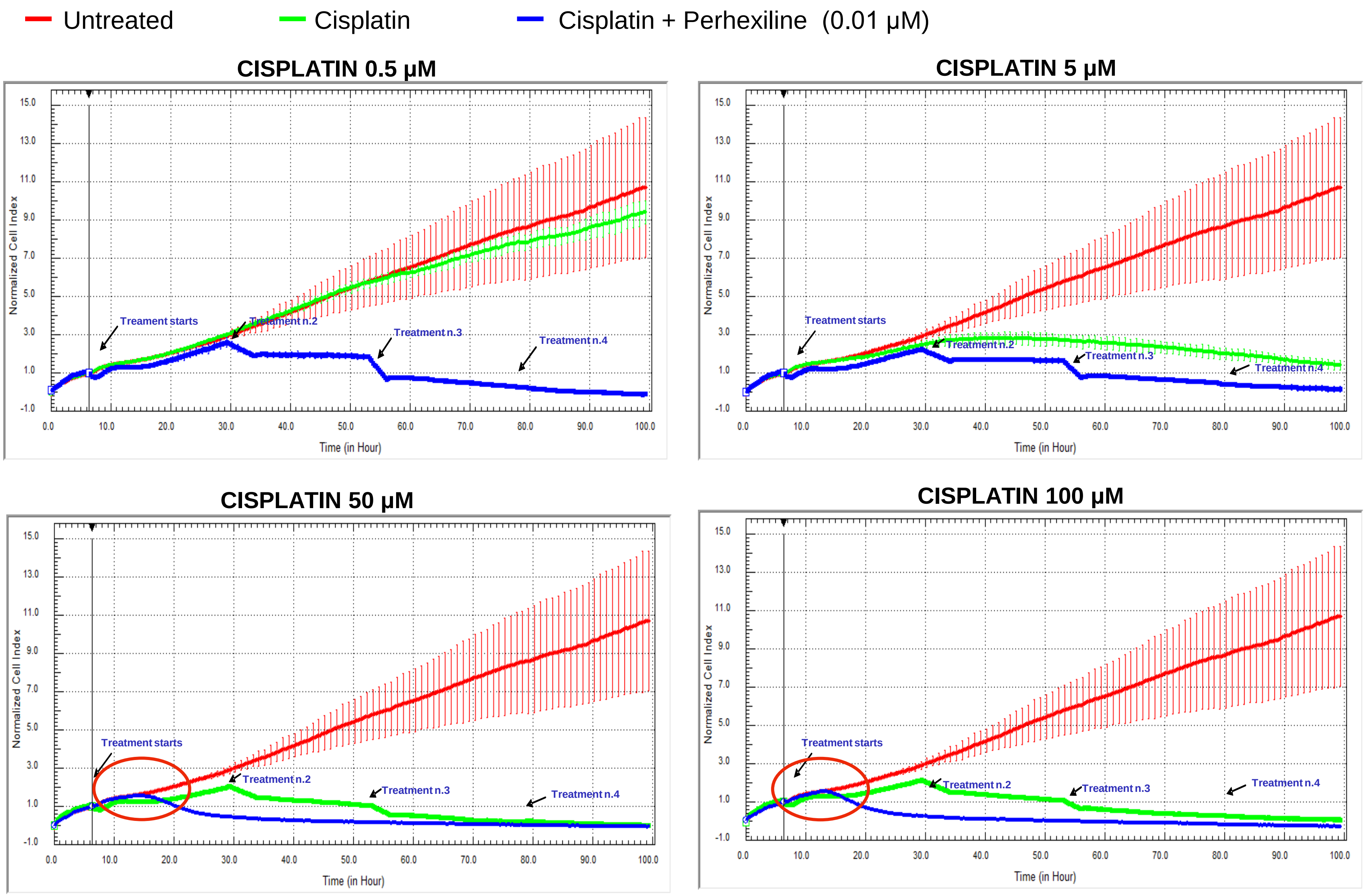

Supplementary Figure S6. Dynamic monitoring of perhexiline effect (0.01 μM), as single treatment (**a**) or daily treatment (**c**), or cisplatin effect (**b** and **d**) (0.5-5-50-100 μM) on the viability of SH-SY5Y wt was measured based on the dose–response curves of the cell index by the xCELLigence system. CI was recorded every 30 minutes. Each trace at each concentration was an average of at least two replicates.

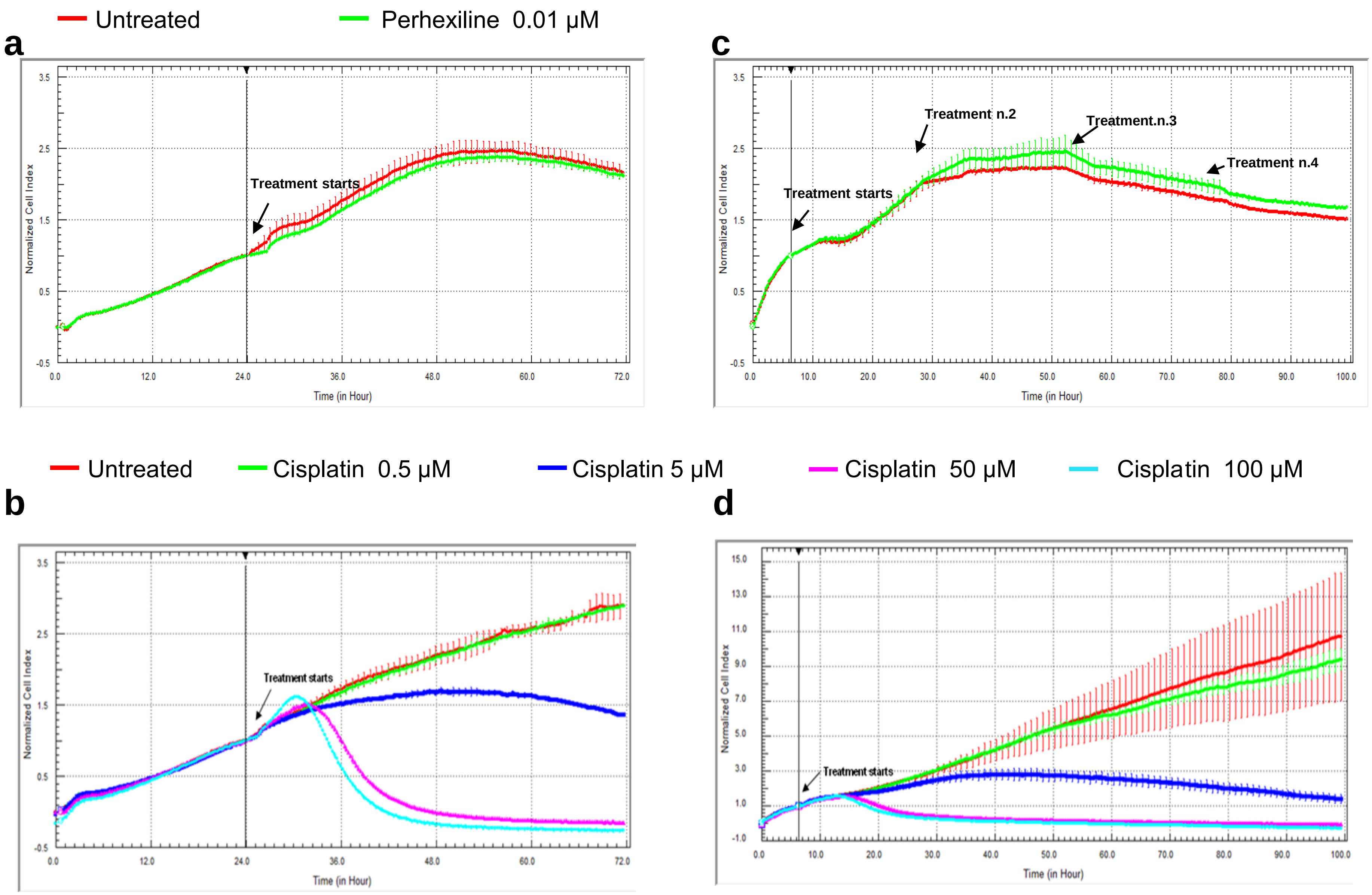

Supplementary Figure S7. **a**) Real-time RT-PCR detection NDM29 mRNA amount in permanently transfected cell lines: SH-SY5Y Mock cells, transfected with pEGFPN1 plasmid, which express NDM29 at its basal level (left panel); SHSY-5Y AntiNDM29 cells, in which NDM29 RNA level was decreased by 30% (righ panel). Values are mean ± SD. One star (*) indicates $p<0.05$, two-tailed Student's test.
b) Dynamic monitoring of cisplatin effect (0.05-0.5-5 μM) on the viability of SH-SY5Y wt cells permanently transfected with pEGFPN1 plasmid (left panel) and pEGFPN1-siNDM29 (right panel). The cisplatin effect was measured based on the dose–response curves of the cell index by the xCELLigence system. CI was recorded every 30 minutes. Each trace at each concentration was an average of at least two replicates.

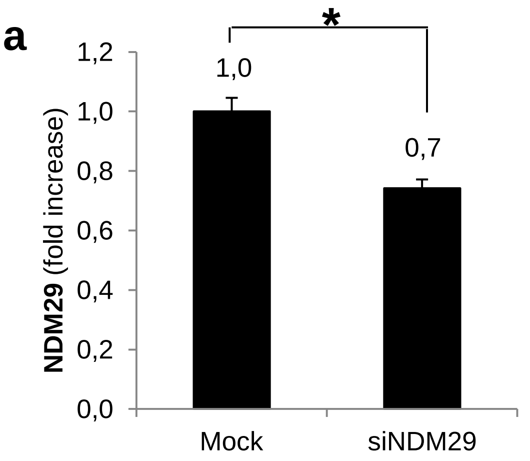

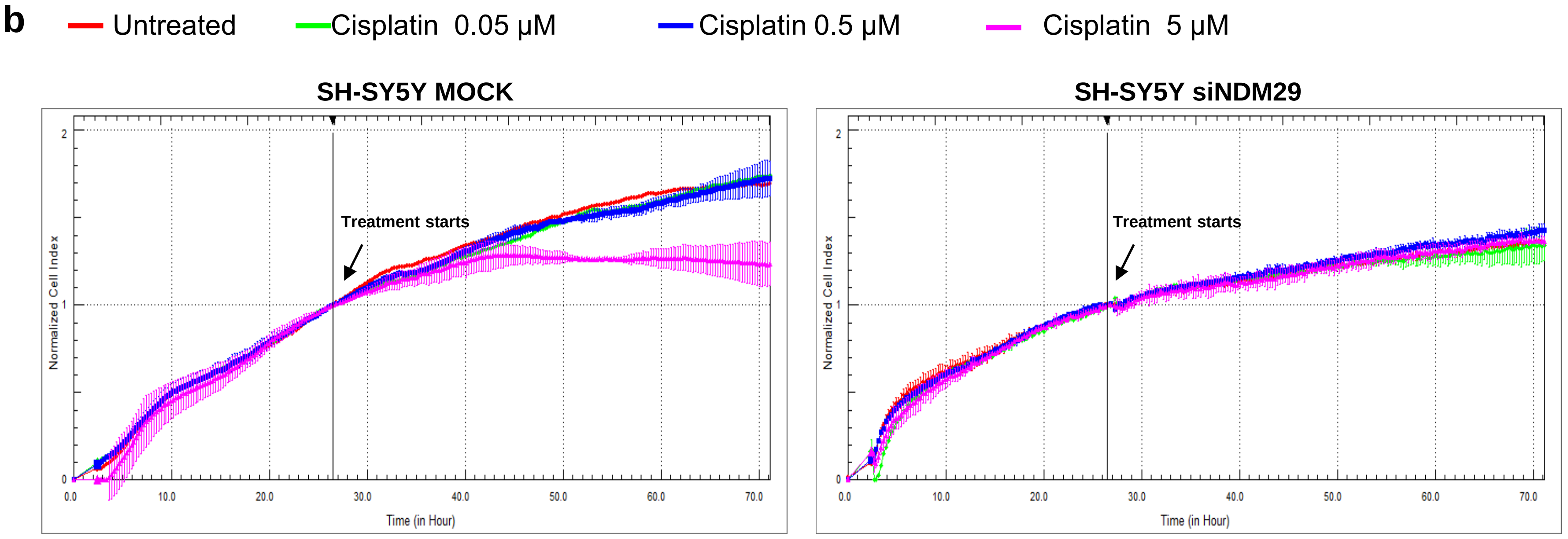

Supplementary Figure S8. Kinetics of cytotoxicity responses for cisplatin in SH-SY5Y cells permanently transfected (Mock and siNDM29), treated or not with perhexiline (0.01 μM) and cisplatin (0.05-0.5-5 μM), monitored by the RT-CES system. Different compound concentrations are indicated by different colours. CI was recorded every 30 minutes. Each trace at each concentration was an average of three replicates. Data are normalized to the time of compound addition of cell culture. Each trace at each concentration was an average of at least two replicates.

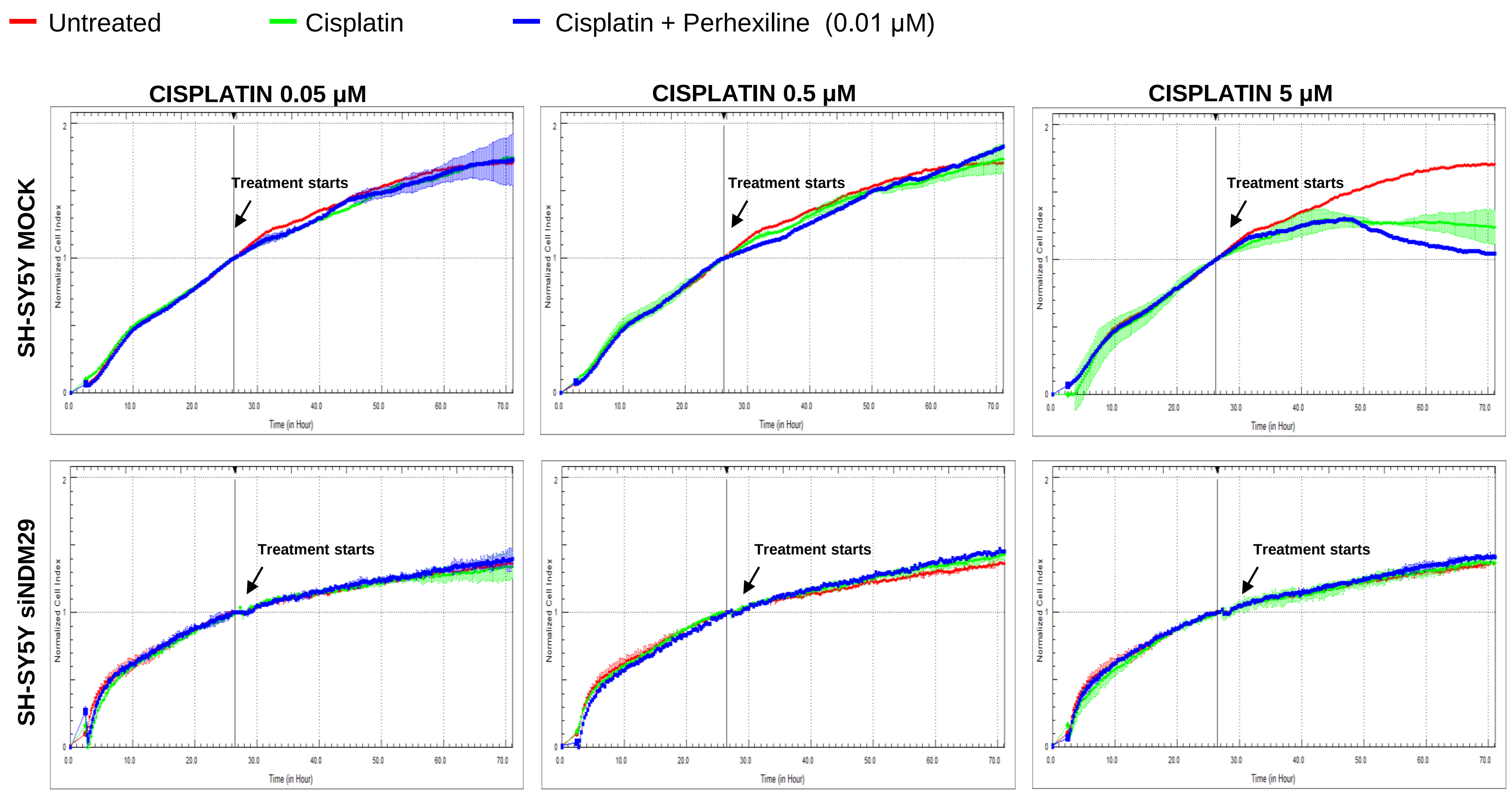

Supplementary Figure S9. **a**) Representative pictures of colonies at day 12 after seeding for each experimental conditions (perhexiline 0.01 μM and/or cisplatin 5 μM) for SH-SY5Y cells permanently transfected (Mock and siNDM29). Magnification 4X. Scale bar: 1000 μm.
b) Capacity of SH-SY5Y cells permanently transfected (Mock and siNDM29) to form colonies in methylcellulose in presence of different treatments (perhexiline 0.01 μM + cisplatin 5 μM *vs.*cisplatin 5 μM. The data are presented as the mean ± SD (n=6 field for each treatment).

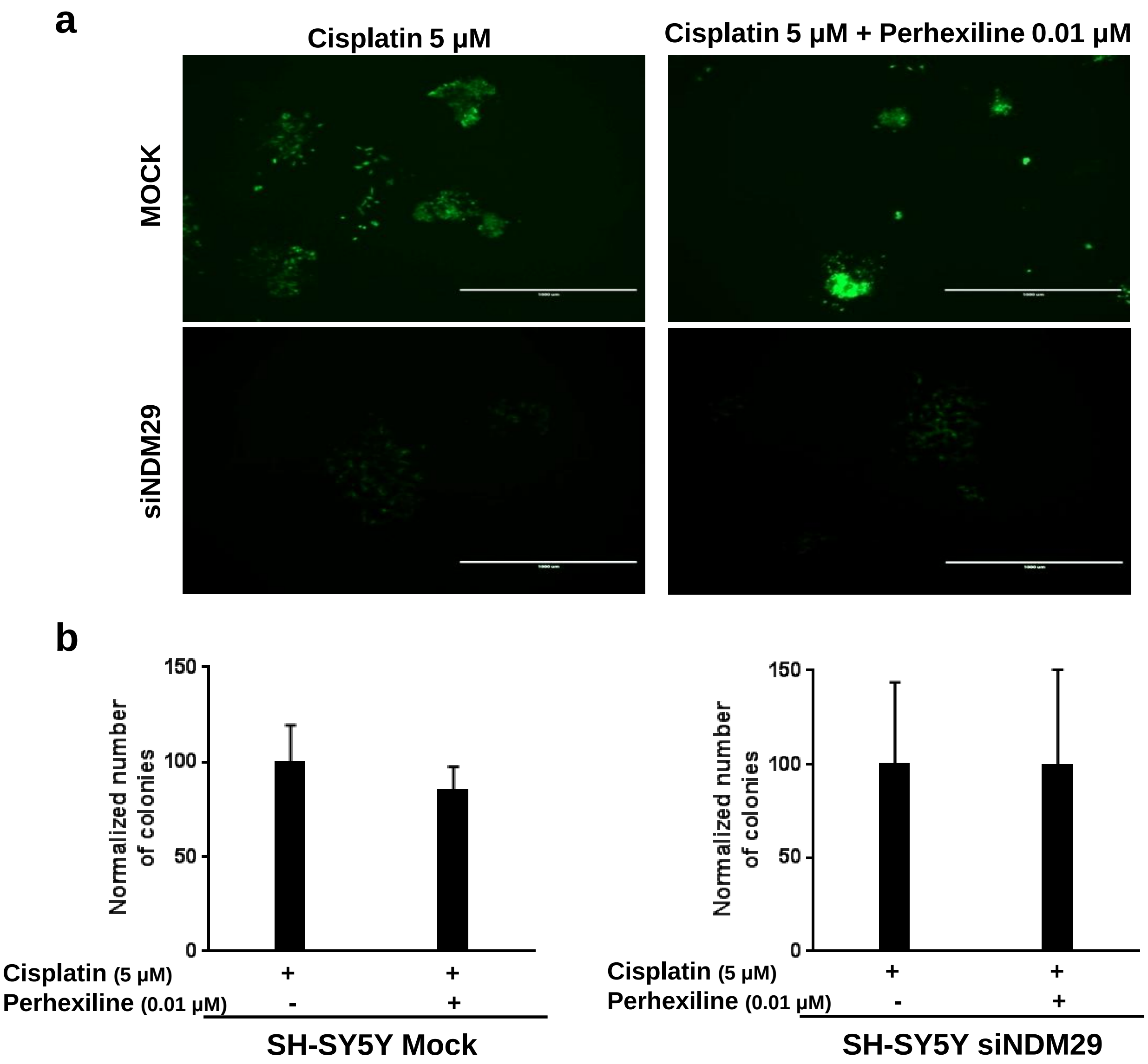

Supplementary Figure S10. Diagrams of FITC-Annexin V/PI flowcytometry of freshly isolated cells from treated and untreated tumors from protocol *a* (**a**) or *b* (**b**). The lower left quadrants of each panel show the viable cells, which exclude PI and are negative for FITC-Annexin V binding. The upper right quadrants (R3) contain the nonviable, necrotic cells, positive for FITC-Annexin V binding and for PI uptake. The lower right quadrants (R5) represent the apoptotic cells, FITC-Annexin V positive and PI negative. One representative experiment for each experimental condition is shown. The averaged percentage of viable, apoptotic and necrotic cells in the considered groups of mice are represented. Protocol *a* (**c**), protocol *b* (**d**).

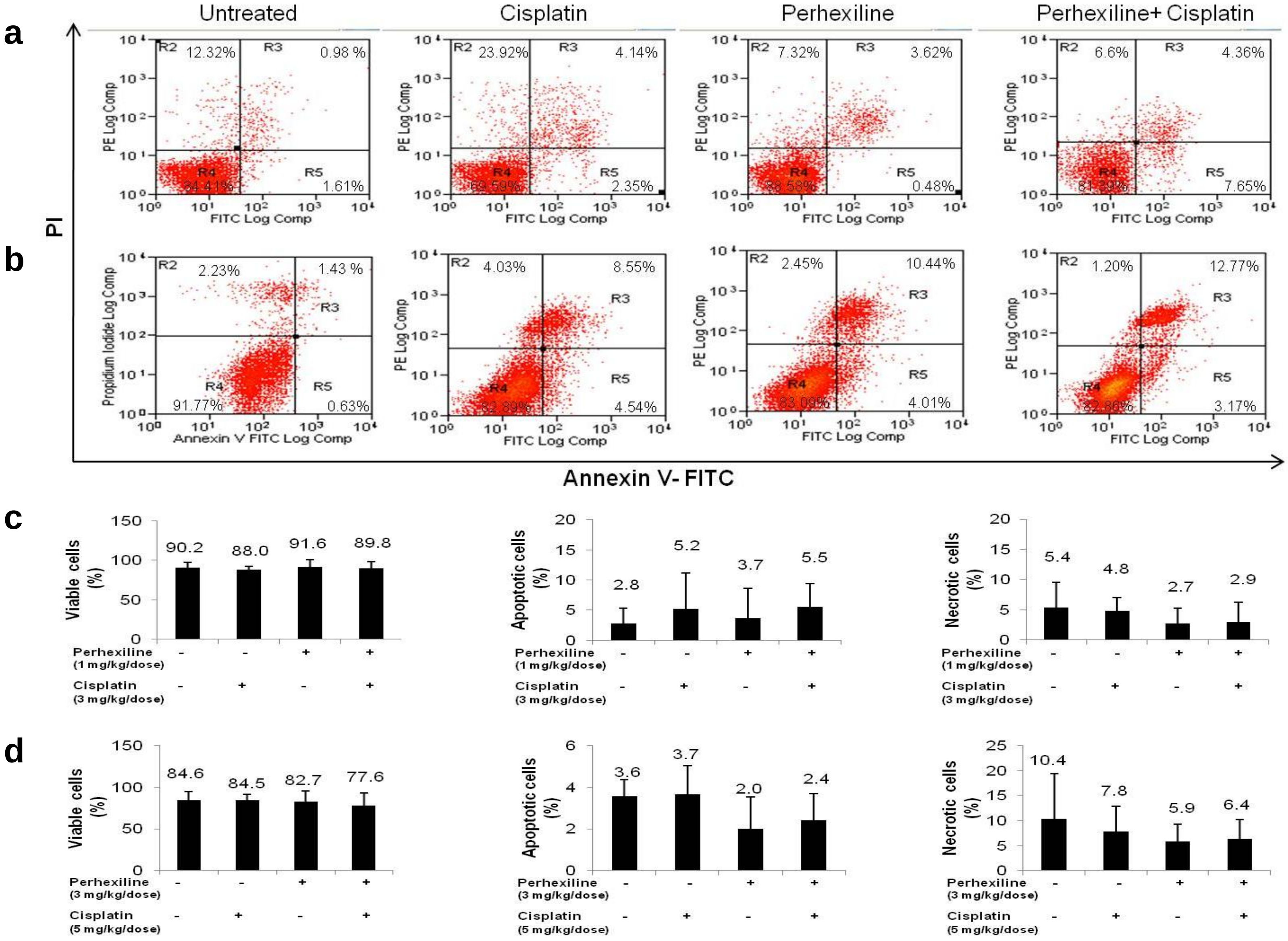

Supplementary Figure S11. Photographs of dissected tumour from treated and untreated mice (protocol *a*).

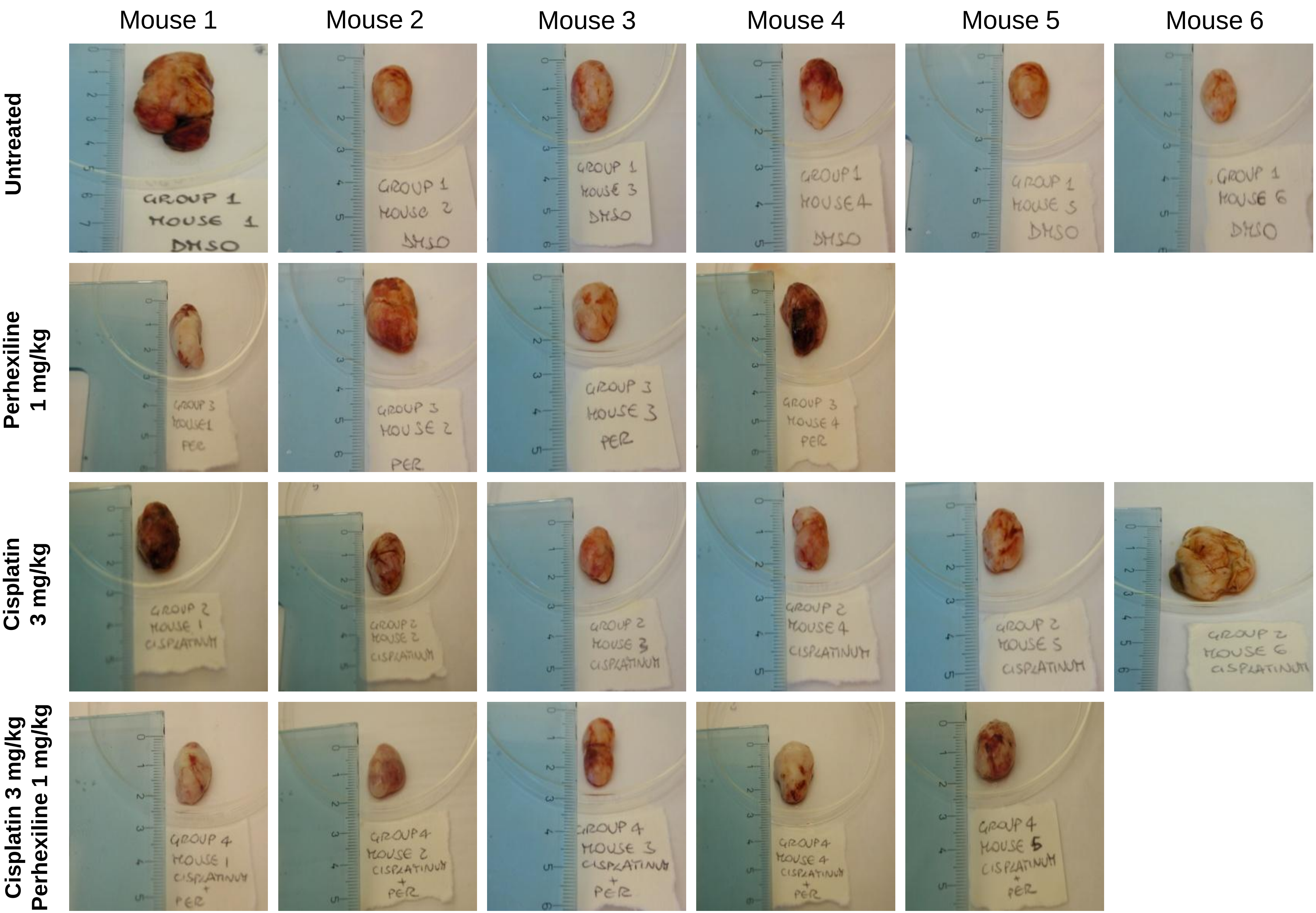

Supplementary Figure S12. Photographs of dissected tumour from treated and untreated mice (protocol *b*).

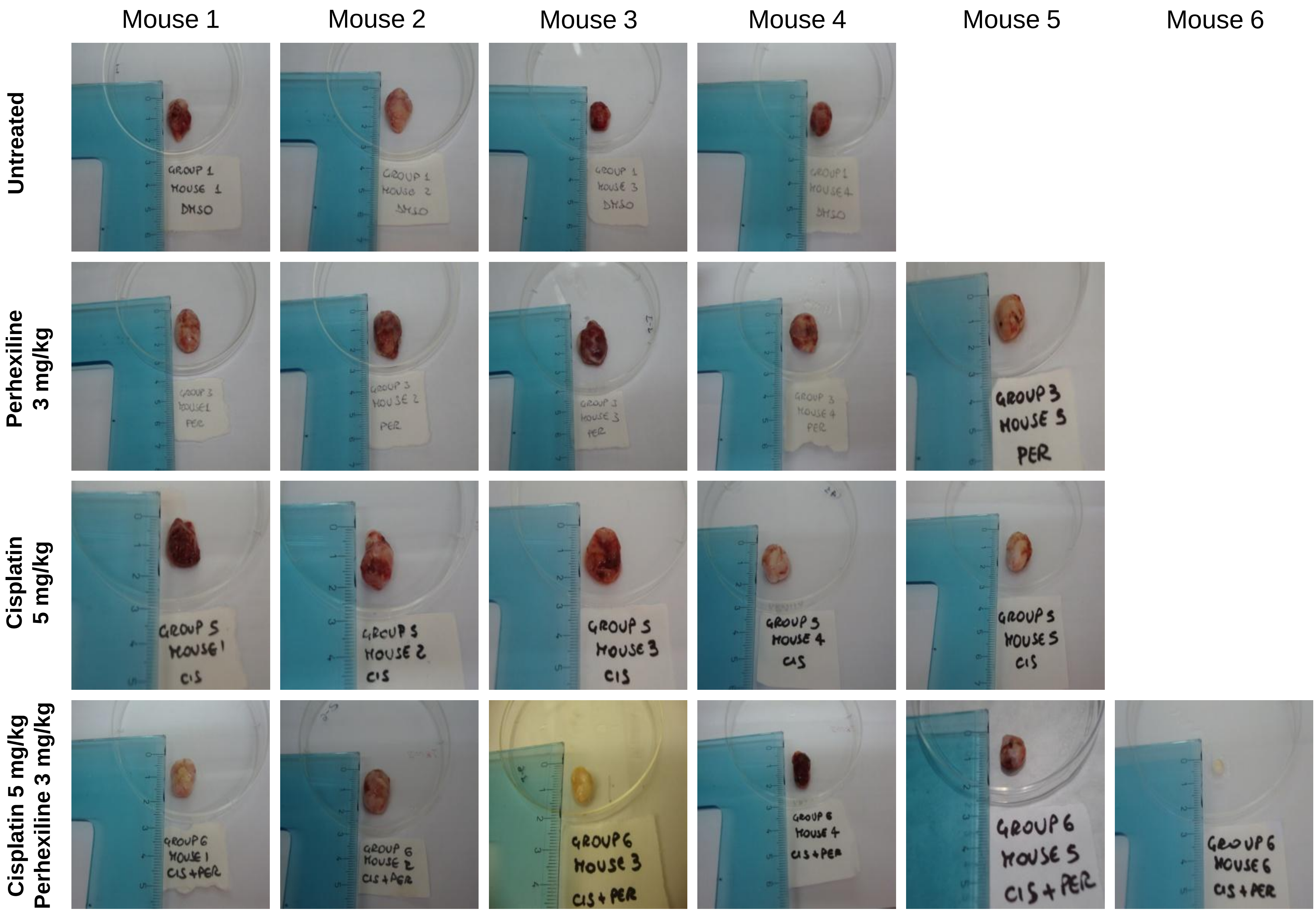

Supplementary Table 1. Oligonucleotide sequences used for gene detection

Gene	Forward primer	Reverse primer
**NDM29**	GGCAGGCGGGTTCGTT	CCACGCCTGGCTAAGTTTTG
**NF68**	CAAGGACGAGGTGTCCGAG	CCCGGCATGCTTCGA
**C-Kit**	GCAAGTCAGTGCTGTCGGAA	AAGATAGCTTGCTTTGGACACAGA
**ABCA1**	GCGAGTACTTCGTTCCAACATG	TCGGGAAGGGAGATGTAGAGTTT
**ABCA12**	ATGCATCTGCCCAGAAGTGTT	GGTGTGTTCATTCGGTTGCTT
**SLC7A11**	TCCATGAACGGTGGTGTGTTT	ACCCTCTCGAGACGCAACAT
**GAPDH**	GAAGGTGAAGGTCGGAGTC	GAAGATGGTGATGGGATTTC
